# Structural insights into the disruption of TNF-TNFR1 signalling by small molecules stabilising a distorted TNF

**DOI:** 10.1038/s41467-020-20828-3

**Published:** 2021-01-25

**Authors:** David McMillan, Carlos Martinez-Fleites, John Porter, David Fox, Rachel Davis, Prashant Mori, Tom Ceska, Bruce Carrington, Alastair Lawson, Tim Bourne, James O’Connell

**Affiliations:** 1 UCB Pharma, Slough, SL1 3WE UK; 2 GlaxoSmithKline, Stevenage, SG1 2NY UK; 3 UCB Pharma, Bainbridge Island, WA 98110 USA

**Keywords:** Tumour-necrosis factors, Mass spectrometry, Extracellular signalling molecules, X-ray crystallography

## Abstract

Tumour necrosis factor (TNF) is a trimeric protein which signals through two membrane receptors, TNFR1 and TNFR2. Previously, we identified small molecules that inhibit human TNF by stabilising a distorted trimer and reduce the number of receptors bound to TNF from three to two. Here we present a biochemical and structural characterisation of the small molecule-stabilised TNF-TNFR1 complex, providing insights into how a distorted TNF trimer can alter signalling function. We demonstrate that the inhibitors reduce the binding affinity of TNF to the third TNFR1 molecule. In support of this, we show by X-ray crystallography that the inhibitor-bound, distorted, TNF trimer forms a complex with a dimer of TNFR1 molecules. This observation, along with data from a solution-based network assembly assay, leads us to suggest a model for TNF signalling based on TNF-TNFR1 clusters, which are disrupted by small molecule inhibitors.

## Abstract (web summary)

Small molecules stabilising a distorted TNF trimer can inhibit TNF signaling, but the underlying mechanism is unclear. Here, the authors characterize the inhibitor-bound TNF-receptor complex structurally and biochemically, showing that the inhibitors alter TNF-receptor binding stoichiometry and cluster formation.

## Introduction

TNF, like most members of the TNF superfamily (TNFSF) exists mainly as a trimer with a threefold symmetry resulting in three identical receptor binding sites^1^. TNF signals through two receptors (TNFR1 and TNFR2) that share a similar structural arrangement with an N-terminal extracellular domain (ECD) composed of four cysteine-rich domains (CRDs), an α-helical transmembrane domain and a cytoplasmic domain^1^. The two receptors are most divergent in the cytoplasmic domain, where TNFR1 has a death domain that is absent from TNFR2 resulting in the alternative signalling pathways observed through the two receptors^2^.

The mechanism by which TNF induces signalling across the membrane is poorly understood, however, it is generally believed that the process involves receptor clustering. Indications towards this concept came from observations that signalling was induced by crosslinking receptors using antibodies specific for the extracellular domain of TNFR1^3–5^. In addition, increasing receptor clustering simply through overexpression also resulted in signalling^6^. The structure of the TNFβ–TNFR1 complex highlighted how the proximity of the three receptors upon ligand engagement was conducive to the idea that ligand binding could transduce clustering across the membrane to the cytoplasmic domains^7^. Clustering of the receptor is also supported by observations that the cytoplasmic death domain of TNF and other TNFSF members can form larger ordered assemblies, and other components of the TNFR1 signalling complex are trimeric^6,8–13^.

Studies have revealed that receptor clustering is not just driven by ligand binding, TNFR1 and other TNFR superfamily (TNFRSF) members are pre-clustered prior to ligand binding through what has been termed the pre-ligand assembly domain (PLAD)^14,15^. The membrane-distal CRD (CRD1) forms the PLAD and the nature of the interaction is illustrated in the dimeric crystal structure of TNFR1 (PDB ID: 1NCF [https://www.rcsb.org/structure/1NCF])^16^. In the 1NCF [https://www.rcsb.org/structure/1NCF] structure the ligand-binding interface (through CRD’s 2 & 3) is on the opposite face of the receptor to the PLAD interaction, an arrangement that lends itself to the building of signalling networks linked by ligand–receptor and receptor–receptor interactions^16^. It is likely that the disruption of such a network by inhibition of one or more receptor binding events could have an impact on TNF signalling.

There are currently five approved TNF biologics that work by completely blocking the interaction of TNF with its receptors but to date, there are no small molecule therapeutics available that disrupt the high-affinity TNF–TNFR interaction. There are reports of small molecule inhibitors that either disrupt the formation of the TNF trimer (SP307 & JNJ525) or distort the trimeric symmetry of another TNF family member, CD40L, by intercalating between monomers of the trimer (BIO8898)^17–19^. Subsequently, compounds (UCB-6786, UCB-5307 & UCB-9260) have also been described that, rather than intercalate between monomers, stabilise a distorted TNF trimer by binding entirely within the core of the trimer, resulting in an asymmetrical TNF trimer capable of binding TNFR1 at only two of the three receptor binding sites^20^.

Using crystallography and solution-based techniques, we further characterise the mechanism of action of the UCB-series of compounds. The crystal structure of compound-bound mouse TNF (mTNF) in complex with humanTNFR1 (hTNFR1) reveals a distorted mTNF trimer with two copies of hTNFR1 bound. Significantly, the bound receptors are dimeric in nature mirroring the PLAD dimer crystal structure (1NCF [https://www.rcsb.org/structure/1NCF]). We also present data from an in vitro network assembly assay which suggests CRD4 of TNFR1 has a role in receptor clustering, highlighting the possibility that a TNFR1 dimer with alternative conformation to those published may be required for the assembly of larger signalling networks.

## Results

### Compounds reduce the affinity of TNFR1 binding at one of the receptor binding sites on TNF

Recently, we described small molecule inhibitors of TNF that stabilised a distorted conformation of human TNF (hTNF) by binding in a pocket within the trimer core^20^. The altered conformation resulted in disruption of one of the three receptor binding sites, restricting the capacity of TNF to bind its receptor (TNFR1) to two copies per trimer^20^. To better understand the impact of these changes on the affinity of receptor binding, we applied a number of solution-based techniques in combination with a selected number of compounds from the same chemical series. Each of the compounds bound hTNF in a similar mode within the trimer core, resulting in an equivalent degree of distortion in the trimer (Supplementary Fig. 1).

**Fig. 1:**
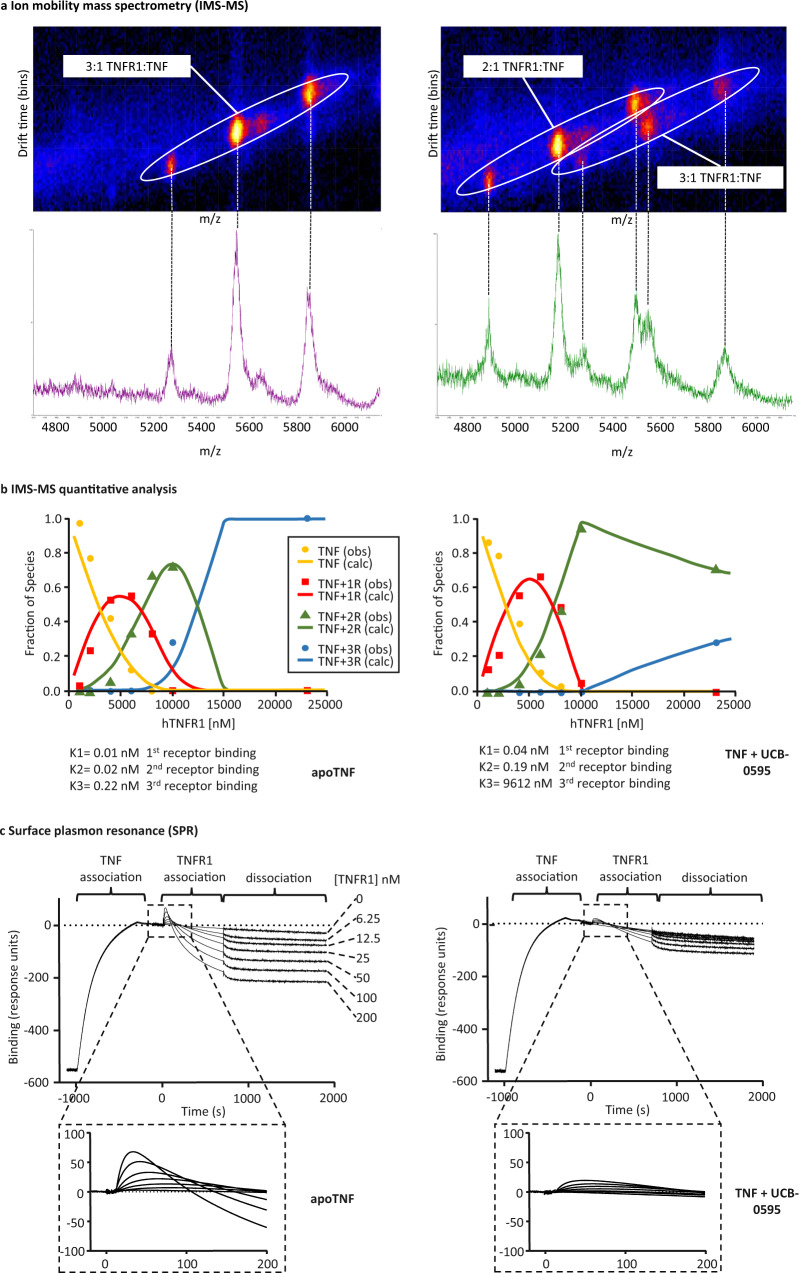
Effect of compounds on TNF–TNFR1 interaction. **a** IMS-MS of hTNF with 5-fold excess hTNFR1 (left panel), hTNF plus UCB-0595 (10-fold excess) and 5-fold excess hTNFR1 (right panel). Circled signals and corresponding peaks show three ionisation states for each receptor-bound form. **b** Quantitative analysis of IMS-MS data generated using hTNF and hTNF plus UCB-0595 (10-fold excess) (left and right panels, respectively) over a range of hTNFR1 concentrations (*x* axis). Traces (calc—calculated) indicating the percentage of each species; 0 receptor, 1 receptor, 2 receptor and 3 receptor-bound are shown (yellow, red, green and blue traces, respectively). Symbols (obs—observed) represent experimentally measured molar fractions of the different species in equilibrium (*n* = 1 independent experiments, a similar reduction in affinity of the third receptor binding was observed for multiple compounds—Supplementary Table 1). Source data are provided as a Source Data file. **c** SPR of hTNF (left panel) and hTNF plus UCB-0595 (10-fold excess) (right panel) binding to immobilised hTNFR1 (first injection) followed by injection of hTNFR1 at a range of concentrations. Detail of the TNFR1 binding response (third receptor binding) is highlighted (dashed box) (*n* = 1 independent experiments).

By applying ion-mobility mass spectrometry (IMS-MS), we were able to separate the various receptor-bound states of hTNF. Initially, an analysis was done with a fixed ratio of TNF to the receptor (5-fold excess hTNFR1 over hTNF trimer) plus and minus compound UCB-0595 (10-fold molar excess over hTNF trimer). In the sample without compound, as expected, the only form observed was TNF with three receptors bound (Fig. 1a, left panel). In the sample with UCB-0595, a second series of peaks was observed that correspond to TNF with two receptors bound (Fig. 1a, right panel). Peaks related to the three receptor-bound forms were also present, suggesting that there is a population of TNF that may have lost compound allowing three receptors to bind or that UCB-0595 is still present and a third receptor can bind with reduced affinity when receptor concentrations are high.

It is generally thought that TNFR1 binds to TNF with an equal affinity at each site, supported by the fact that the structure of hTNF has three identical receptor binding sites and by analogy with the TNFβ-TNFR1 and TNF-TNFR2 structures (PDB ID: 1TNR [https://www.rcsb.org/structure/1TNR] & 3ALQ [https://www.rcsb.org/structure/3ALQ], respectively). However, to our knowledge, there have been no direct measurements of each individual binding event. Measurements of hTNFR1 binding to hTNF are in the low pico-molar range, but these figures are an average of all sites^21,22^. In order to better understand the effect of our compounds on receptor binding, the IMS-MS data were analysed quantitatively, and an algorithm was developed to enable the derivation of dissociation constants for the three receptor-binding events (details in online methods). Data were generated using IMS-MS for samples of hTNF (with and without UCB-0595 – fully occupied) combined with a range of receptor concentrations. Following quantitative analysis, the data were visualised by plotting the molar fraction of all species (TNF with 0, 1, 2 and 3 receptors bound) against the concentration of receptor added (Fig. 1b). As receptor concentration increases, the fraction of TNF with 0 receptors bound drops (Fig. 1b left graph, yellow line) with a concomitant increase in the population with one, then two and finally three receptors bound (Fig. 1b, left graph red, green and blue lines, respectively) until a steady state is reached, with three receptors bound, at the highest receptor concentrations. After data analysis, equilibrium constants were calculated for the three receptor-binding events. In the absence of compound, binding of the first and second receptors was in the range of 10–20 pM, in line with published average measurements. Unexpectedly, the third receptor binding event appeared to be weaker with a *K*_D_ value ~10-fold higher than the first two (Fig. 1b, left graph, *K* values). This observation suggests that there is a degree of cooperativity involved in receptor binding which needs to be taken into account when considering the mechanism of TNF signalling.

Measurements made in the presence of UCB-0595 revealed a major effect on the third receptor binding event where a shift from 0.22 nM to 9.6 μM was observed (Fig. 1b, right graph, *K* values). A number of compounds have been tested using this method and the change in *K*_D_ was consistently in the region of 5 orders of magnitude greater in the presence of compound (Supplementary Table 1).

To support the observations made by IMS-MS, we attempted to measure the effect of UCB-0595 on the affinity of receptor binding using surface plasmon resonance (SPR). It is recognised that generating accurate affinity measurements of TNFSF ligands for their receptors using SPR is challenging due to the trivalent nature of the interaction^23^. Given that our compounds appear to have the greatest impact on the third receptor binding, an SPR experiment was designed to measure this event. TNFR1 was immobilised on a CM5 sensor chip surface at a density that, following injection of TNF at a high concentration, resulted in a stable TNFR–TNF complex where the receptor binding sites on TNF were not fully saturated (mix of three and two receptors bound). The impact of the compound on the capture of a third receptor could then be measured.

In the absence of compound, titration of soluble TNFR1 resulted in a ternary complex as the third receptor-bound, however, complete binding was not observed due to accelerated dissociation during the association phase (Fig. 1c, left panel). The increased dissociation could be due to soluble receptor competing for binding with the immobilised receptor resulting in displacement TNF from the sensor chip. This dissociation in the association phase prevented the measurement of the affinity of the third receptor binding.

When the experiment was done using TNF saturated with UCB-0595 there was decreased receptor binding and a reduced receptor association rate compared to TNF alone (Fig. 1c, compare zoomed detail left and right panels), confirming the observations made by IMS-MS. Again, dissociation during the TNFR1 association phase was observed preventing the calculation of an affinity constant. Interestingly, the destabilisation of the TNFR1–TNF–UCB-0595 complex was greatly reduced when compared to that in the absence of compound (Fig. 1c compare left and right panels TNFR1 association phase). Indicating that the TNFR1–TNF–UCB-0595 complex was more stable and resistant to disruption by soluble TNFR1.

We have demonstrated that our compounds stabilise the TNF trimer^20^, and the SPR data suggested that the stabilised conformation results in increased affinity for either the first or second receptor-binding events, however, no such effect was observed by IMS-MS. Both techniques highlighted how the compound stabilised, distorted TNF had a significant impact on the third receptor binding, corroborating previously published size exclusion chromatography (SEC) data^20^.

### Structure of compound-bound mouse TNF in complex with two humanTNFR1 dimers

**Fig. 2:**
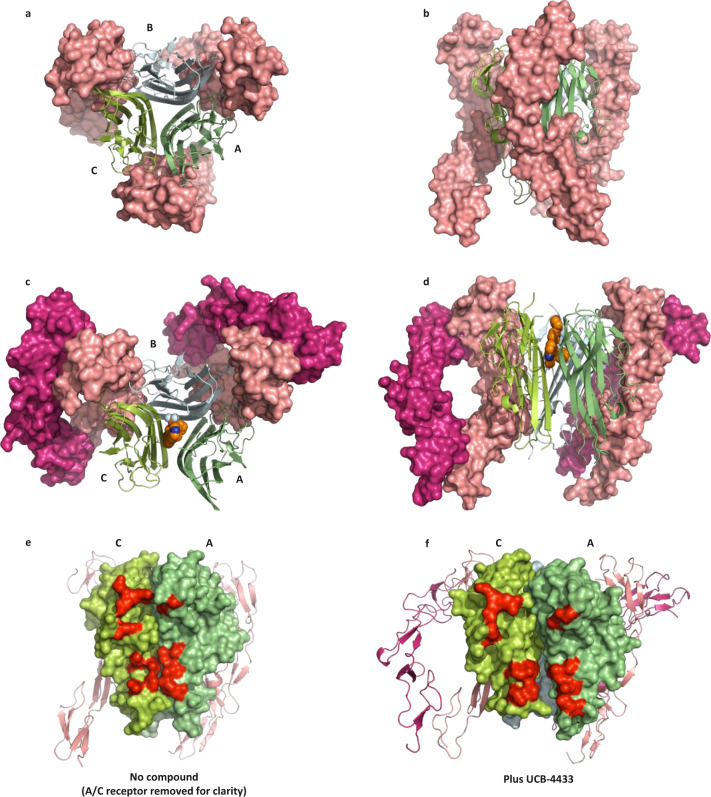
Comparison of mouse TNF-humanTNFR1 complex with and without compound. Top view (**a**) and side view (**b**) of trimeric mTNF (green ribbons, monomers assigned A, B and C) with three copies of monomeric receptor-bound (pink surface rendered) (PDB: 7KP7 [https://www.rcsb.org/structure/unreleased/7KP7]). Top view (**c**) and side view (**d**) of trimeric mTNF (green ribbons) with UCB-4433 bound (orange space-fill) and two copies of dimeric hTNFR1 bound (pink and purple surface rendered) (PDB: 7KP8 [https://www.rcsb.org/structure/unreleased/7KP8]). **e** Side view of mTNF (green surface rendered) without compound—one copy of the hTNFR1 has been removed to reveal the non-distorted a–c receptor binding site (selected residues involved in hTNFR1 binding highlighted in red). **f** Side view of mTNF (green surface rendered) with UCB-4433 bound (not visible) revealing the distorted A–C receptor binding site (selected residues involved in receptor binding highlighted in red).

The effect of our compounds on hTNFR1 binding to hTNF in solution showed a clear reduction from three receptors to two which is consistent with the distorted nature of the compound-bound hTNF (PDB code: 6OOY [https://www.rcsb.org/structure/6OOY])^20^. In order to further understand the impact of our compounds on receptor binding, we have crystallised mTNF with and without compound (UCB-4433) in complex with a truncated form of hTNFR1 (residues 41–184). All attempts to crystallise the complex made with a more complete receptor (residues 41–201) failed as did attempts to crystallise a complex of human TNF with humanTNFR1 regardless of the receptor length. Diffraction data from mTNF–hTNFR1 and mTNF(UCB-4433)-hTNFR1 crystals were obtained at 3.15 and 2.65 Å resolution, respectively. The structures were then solved by molecular replacement using Phaser with input models based upon previously solved and unpublished crystal structures of hTNF apo and bound with compound and an uncomplexed hTNFR1. In the complex without compound, trimeric TNF adopts a symmetrical threefold symmetry with a monomeric receptor occupying each of the receptor binding sites (Fig. 2a, b) (PDB: 7KP7 [https://www.rcsb.org/structure/unreleased/7KP7]). The individual components, mTNF and hTNFR1, align very closely with published structures (PDB ID: 1TNF [https://www.rcsb.org/structure/1TNF] and 1NCF [https://www.rcsb.org/structure/1NCF], root-mean-square deviation (RMSD) of 0.32 and 0.98 Å, respectively), and the overall tertiary structure is very similar to the TNFβ–TNFR1 complex (1TNR [https://www.rcsb.org/structure/1TNR], RMSD of 3.01 Å). In agreement with the structure of hTNF plus UCB-6876 (PDB ID: 6OOY [https://www.rcsb.org/structure/6OOY])^20^, UCB-4433-bound mTNF in complex with receptor was similarly distorted (PDB: 7KP8 [https://www.rcsb.org/structure/unreleased/7KP8]), (comparing compound-bound forms of mTNF and hTNF gave an RMSD of 0.63 Å). In order to strengthen the correlation between structural observations made with the mTNF(UCB-4433)–TNFR1 complex and how they are likely to also apply to hTNF, a comparison of the binding mode of UCB-4433 in human and mouse TNF was made. Comparing the mTNF(UCB-4433)–TNFR1 (PDB: 7KP8 [https://www.rcsb.org/structure/unreleased/7KP8]) and hTNF(UCB-4433) (PDB: 7KP9 [https://www.rcsb.org/structure/unreleased/7KP9]) structures clearly highlighted how the mode of compound binding was highly conserved (Supplementary Fig. 2).

The degree of disruption of one of the receptor binding sites in the mTNF(UCB-4433)-TNFR1 structure was such that only two receptors were bound (Fig. 2c, d). Significantly, receptors that were bound were dimeric (described in detail below). In order to highlight the degree of distortion of the, arbitrarily defined, A–C receptor binding site, the UCB-4433 bound mTNF–hTNFR1 structure was compared with the complex without compound by aligning them through monomer C (Fig. 2e, f). Monomer A is twisted and tilted down and away from monomer C resulting in their partial separation. This is more clearly illustrated towards the bottom of the receptor-binding site where the gap between the residues highlighted in red is clearly widened (Fig. 2f). The degree of shift was measured for a number of amino acids that are involved in TNFR1 binding and the displacement ranged from 6.2 to 7.8 Å (Supplementary Table 2), making it impossible for the receptor to bridge across and bind the two monomers simultaneously, as observed at the two other binding sites.

The individual monomers maintain their fold upon compound binding (RMSD of 0.5 Å), so the relevant positioning of residues involved in receptor binding within each individual monomer remains largely unchanged. It is, therefore, possible that receptor could still bind (albeit weakly) to either monomer A or C, however, this was not observed in the crystal structure suggesting that receptor binding prefers contact with adjacent monomers in the trimer to form a stable interaction.

**Fig. 3:**
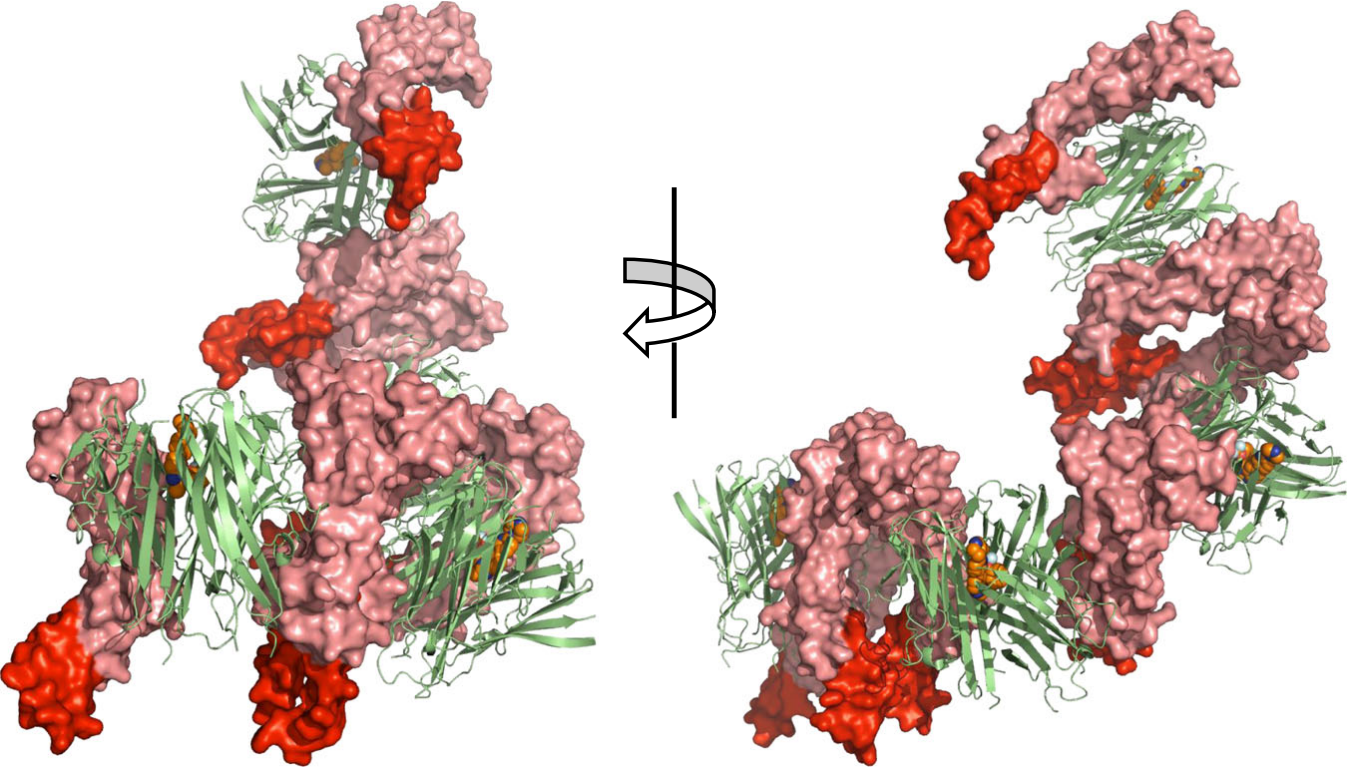
Extended TNF–TNFR1 chain. Four copies of trimeric mTNF (green ribbons) linked by hTNFR1 dimers (pink surface rendered) as present in the crystal lattice (PDB: 7KP8 [https://www.rcsb.org/structure/unreleased/7KP8]). Membrane proximal ends of each receptor are highlighted in red.

A key feature of the structure presented is the dimeric nature of the receptors (Fig. 2c, d). The arrangement of the receptor dimer matches that of the published hTNFR1 receptor dimer in isolation (1NCF [https://www.rcsb.org/structure/1NCF], RMSD of 1.4 Å), an arrangement that is conducive to the multimerization of the TNF trimer–receptor complex. Indeed, when additional copies of the ligand–receptor complex within the crystal lattice were located it was clear that an extended TNF–receptor chain had formed, however, because there is a turn of ~35° between adjacent trimers the chain adopts a curved spiral arrangement (Fig. 3).

### Soluble hTNF and hTNFR1 auto assemble into large insoluble networks

The mTNF–hTNFR1 structures described above were generated using a truncated form of hTNFR1 (41–184), missing half of CRD4, as initial attempts to achieve this with a more complete receptor, containing all four CRD’s (residues 41–201), resulted in large amounts of protein precipitation when the proteins were mixed. Lowering the protein concentration failed to prevent the precipitation which was unexpected given that individually hTNF and hTNFR1(41–201) were soluble at high concentrations (10 mg/mL). Intrigued by these observations, we decided to further characterise the nature of the aggregation event.

**Fig. 4:**
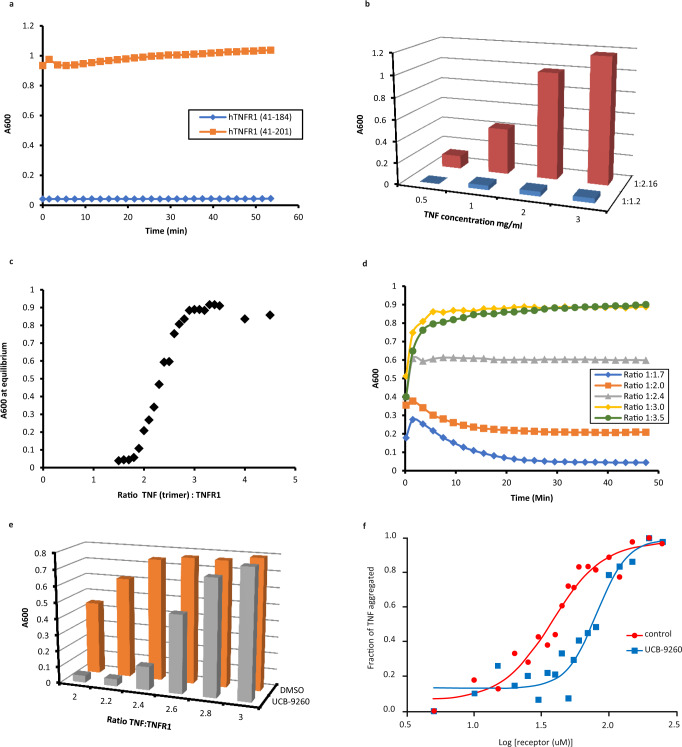
In vitro TNF–TNFR1 network assembly. **a** Aggregation of hTNF (3 mg/mL) +hTNFR1(41–201) (orange trace) and hTNF (3 mg/mL) +hTNFR1(41–184) (blue trace) over time (ratio of TNF:TNFR = 1:2.16). **b** Aggregation of hTNF +hTNFR1(41–201) over a range of concentrations and at two ratios of hTNF:hTNFR1 (1:1.2 blue bars and 1:2.16 red bars). **c** Aggregation state of hTNF (2 mg/mL) +hTNFR1 at equilibrium over a range of ratios. **d** Aggregation of hTNF (2 mg/mL) +hTNFR1 at five ratios (1:1.7 blue trace, 1:2 orange trace, 1:2.4 grey trace, 1:3 yellow trace, 1:3.5 green trace) over time. **e** Aggregation of hTNF (2 mg/mL) + hTNFR1 over a range of ratios with UCB-9260 (10-fold excess over hTNF trimer) (grey bars) and DMSO only (orange bars) (*n* = 1 independent experiments). **f** Aggregation of hTNF +hTNFR1 at a fixed ratio of TNF:TNFR = 1:3.2 over a range of total protein concentrations with UCB-9260 (5-fold excess over TNF) (blue trace) (*n* = 1 independent experiments, a similar rightward shift in the point of aggregation was observed for multiple compounds—Supplementary Fig. 3) and DMSO control (red trace) (*n* = 2 independent experiments). Source data are provided as a Source Data file.

To confirm the difference between the long (41–201) and short (42–184) forms of the receptor, hTNF was mixed with each form and precipitation was monitored over time. There was no evidence of precipitation with the short form whereas the long form precipitated very rapidly reaching almost maximal signal in the seconds taken to transfer the plate to the spectrophotometer for reading (Fig. 4a).

Next, we investigated the effect of protein concentration and the ratio of TNF:TNFR1. There was a clear dependence on the ratio of the proteins, with no evidence of precipitation at a ratio of 1:1.2 (trimer:receptor) regardless of the total protein concentration used (Fig. 4b, blue columns). At a ratio of 1:2.16 precipitation was observed at all concentrations, the increase in signal with increasing TNF concentration was mainly due to there being more protein to precipitate, therefore a higher A_600_ was observed (Fig. 4b, red columns). These data suggest that the aggregation is driven by the TNF:TNFR1 ratio rather than overall protein concentration. To try and understand the exact point at which aggregation was triggered, a finer sampling of the TNF:TNFR1 ratio was made (Fig. 4c, d). In samples that had reached equilibrium, there was very little if any detectable aggregation at ratios below 1:2 but as soon as this point was passed, aggregation increased in a linear fashion directly related to the amount of receptor used (Fig. 4c). Once the ratio had reached a point where all binding sites on TNF were fully occupied with the receptor (1:3 and above) the degree of aggregation levelled off. These data clearly suggest that the trigger for aggregation was related to the binding of the third receptor and the fact that aggregation peaked once TNF was fully occupied by receptor confirms that the aggregation was not caused by a non-specific ligand–receptor interaction.

In the above study, as in previous experiments, aggregation occurred as soon as the two proteins were mixed and progressed to a state of equilibrium over a period of ~30 min (Fig. 4d). At ratios of 1:2 and below there was a noticeable increase in the degree of aggregation in the first minute or so after mixing the proteins (A_600_ spike around 2 min in the 1:1.7 and 1:2 samples, Fig. 4d), followed by a levelling off over time. We believe this is due to a brief localised concentration of receptor that momentarily pushes the ratio above 1:2 resulting in aggregation. As the sample fully mixes the aggregate dissolves reaching equilibrium by 30 min.

From these data, we propose that the aggregate formed is not a random, non-specific agglomeration of protein but is in fact an ordered protein network, stabilised by specific TNF–TNFR1 and TNFR1–TNFR1 interactions and may well represent the signalling networks that are believed to form on the cell surface. In addition, the trigger for forming the network is binding of the third receptor and interestingly, precipitation only occurred when using TNFR1 with an intact CRD4. The fact that the short, PLAD containing, receptor did not form networks suggests that an alternative, as yet, uncharacterised receptor dimer had formed with the long receptor, one that may involve CRD4.

### Inhibition of network assembly through compound binding to TNF

Given that our compounds reduced the affinity of the third receptor binding, which from the data presented above, appears to be a critical step in the assembly of TNF–TNFR1 networks, we anticipated that they would inhibit the aggregation/network assembly process. To address this question, the degree of aggregation was measured for hTNF (± UCB-9260) at a range of ratios with the receptor. At lower ratios, UCB-9260 completely blocked aggregation delaying the onset until a ratio of 1:2.4 was reached, at which point the DMSO control sample was close to a maximum state of aggregation (Fig. 4e). At higher ratios UCB-9260 was unable to block the aggregation process, suggesting that three receptors had bound triggering the receptor–receptor contacts required for a network to form.

Network assembly is dependent on both ligand–receptor and receptor–receptor interactions and it is possible our compounds could influence both. Having demonstrated the impact on ligand–receptor interaction, an alternative aggregation assay was developed to better understand how compounds affect the receptor–receptor interaction.

In this assay, the ratio of TNF:TNFR1 was kept constant at 1:3.2, ensuring TNF was fully occupied with three receptors, a state that results in aggregation. To control aggregation under these conditions, the concentration of the TNF–TNFR1 complex was varied. At very low concentrations of complex, very little, if any, aggregation occurred probably because the concentration was below the *K*_D_ of the receptor–receptor interaction. As the concentration of the complex was increased, aggregation was observed, driven by the receptor–receptor interaction (Fig. 4f, red line). Using this method, the effect of UCB-9260 on the receptor–receptor binding in the network could be analysed. In the presence of UCB-9260, higher concentrations of the complex were required to trigger aggregation, indicated by a shift in the curve to the right (Fig. 4f, blue line). As the concentration was further increased aggregation was observed, indicating that UCB-9260 had reduced the affinity of the receptor–receptor interaction, but not blocked it completely. Alternatively, it is possible that at higher concentrations compound is displaced from the complex allowing aggregation to occur. A number of compounds have been tested using this method and the impact on aggregation was consistent (Supplementary Fig. 3).

**Fig. 5:**
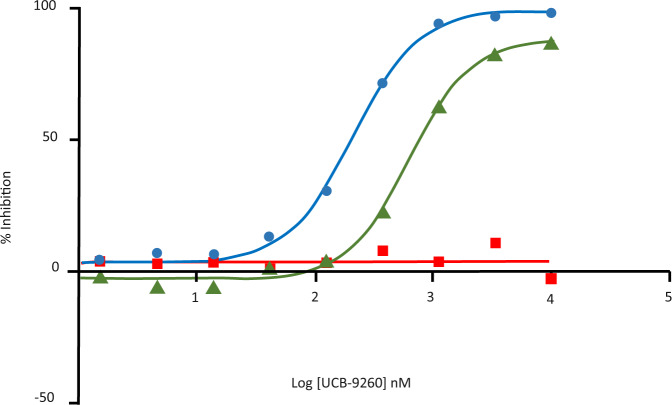
Inhibition of TNF signalling in cells. Representative concentration–response curves showing the effect of UCB-9260 on signalling in an NFκβ reporter cell assay driven by 10 pM hTNF (blue trace), 100pM hTNF (green trace) and anti-TNFR1 antibody at 300 ng/mL (red trace). Source data are provided as a Source Data file.

These data demonstrate how at higher TNF–TNFR1 concentrations the inhibitory effect of our compounds, in solution, is reduced. In order to investigate the impact of TNF concentration in cells, we utilised an NFκβ reporter assay previously used to measure the inhibition of TNF siganlling^20^. The reporter assay was run under standard conditions (10 pM hTNF) and at 100 pM hTNF (significantly higher than the sub-10 pM levels of TNF present in the synovium of rheumatoid arthritis patients). At the higher TNF concentration, the potency and efficacy of UCB-9260 were reduced; geometric mean IC50 of 552 nM (*n* = 5, range 407–703 nM) compared to geometric mean IC50 of 208 nM (*n* = 8, range 139–329 nM) when lower levels of TNF were used as indicated by the shift in the curve to the right (Fig. 5). When TNFR1 proximal signalling was assessed, 500 pM TNF had to be used for these assays, and although UCB-9260 was able to significantly inhibit TNFR1 proximal signalling, some residual signalling activity was still observed^20^. These data support the view that if our TNF small molecules are faced with very high levels of TNF–TNFR1 complex, residual signalling may still be observed.

## Discussion

Small molecule inhibitors of TNF are described that stabilise a distorted TNF trimer, reducing the affinity of TNFR1 at one binding site and effectively reducing the stoichiometry of receptor binding from three to two per TNF trimer. It was clear from the TNF–TNFR1 crystal structure that UCB-4433 had prevented a third receptor binding, and studying the effect of compounds in solution suggested that they had significantly reduced the affinity of the third receptor binding. In order to understand the implication of this on TNF signalling, we first need to consider current models of TNF signalling.

Although not fully resolved, it is generally understood that TNF binds to pre-clustered TNFR1 dimers (dimers formed through PLAD) resulting in the formation of a larger signalling network^14–16,24,25^. However, the extended TNF–TNFR1 chain formed with PLAD dimers is twisted into a spiral (Fig. 3). As indicated by the positioning of the membrane-proximal end of the receptors (Fig. 3 highlighted in red), such a conformation is not obviously compatible with the surface of a cell. This indicates that the TNFR1 PLAD dimer may not represent the dimer form present in signalling networks on the cell. In order to generate a network that would be accommodated by the planer nature of the cell surface, the PLAD receptor dimer would need to undergo a conformational change. The need for such a conformational change has been postulated based on a model of an extended TNF–TNFR1 chain that closely matches the structure described here^26^. It was proposed that the energy required to drive a change in conformation of the PLAD dimer could arise from a combination of the force imposed by the high affinity of the ligand–receptor interaction as it tries to assemble into an extended network, along with the force of resistance from the membrane-bound receptor to remain planar^26^. However, our aggregation studies suggest that organised networks can form in the absence of any force of resistance coming from receptor anchored to a membrane and that a PLAD dimer is not sufficient to drive network assembly. In solution it appeared that the trigger for network assembly was binding of the third receptor, specifically a form of a receptor with an intact CRD4, raising the possibility that an alternative receptor dimer conformation that requires CRD4 is key to network assembly.

**Fig. 6:**
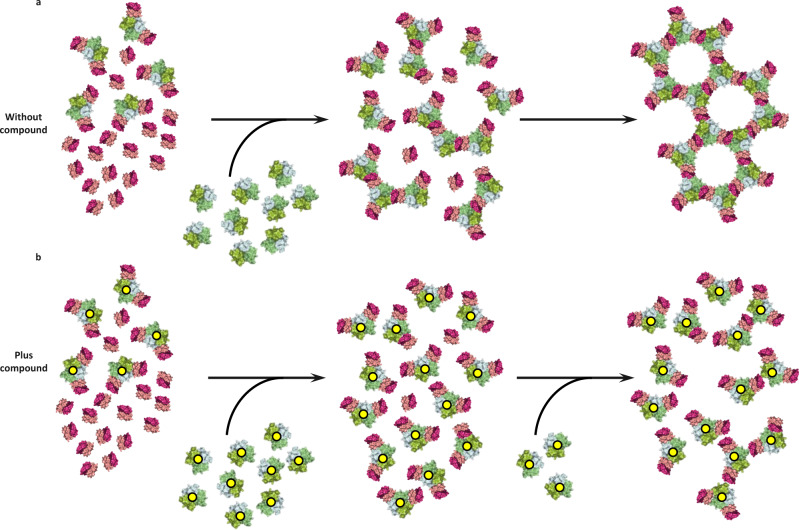
Model of TNF–TNFR1 signalling network. **a** Schematic representation of TNFR1 dimers (pink and purple) pre-clustered, with a portion bound to TNF (green) (two copies of TNFR1 dimer bound per TNF trimer). Addition of more TNF (green) results in linking of the TNF-two-receptor-bound units into larger TNF-three-receptor-bound clusters finally assembling into a large network. **b** Same scenario as **a** in the presence of compound (yellow dots), resulting in incomplete network assembly.

The fact that there was no evidence of aggregation with the short form of the receptor (even when three receptors were bound) suggests that in solution there were no receptor–receptor contacts forming even though this form contains the PLAD. There was also no aggregation when two copies of the long form of receptor were bound, again suggesting there were no receptor–receptor contacts formed. Aggregation only occurred when a third receptor bound, suggesting that this binding event resulted in a conformational change in all three receptors triggering the formation of receptor dimers and assembly of a network. Alternatively, it may be that larger stable networks only form when TNF has three receptors bound, allowing the network to grow in two dimensions through receptor–receptor contacts. On the cell surface it is believed that TNFR1 exists as a dimer^25^; based on this and the observations reported here, we propose a model of TNF signalling (Fig. 6a). Receptor dimers (PLAD dimers?) pre-cluster (pink and purple structures), at low TNF levels (green structures), the first binding events occur through the higher affinity first and second receptor binding sites resulting in receptor dimer–TNF–receptor dimer mini complexes. Such complexes are however unable to grow due to the unfavourable (non-planar) arrangement of the PLAD dimer. With increasing TNF concentrations, the mini complexes accumulate until a critical concentration is reached whereby the third, weaker, receptor binding occurs, inducing a conformational change that results in coupling of the mini complexes into larger signalling networks. Addition of a compound (Fig. 6b, yellow dots) to such a model would prevent binding of the third receptor until TNF and receptor concentrations were sufficiently high to drive the binding of a third receptor at the distorted site. Any receptor able to bind at this site would do so in a sub-optimal way resulting in a distorted signalling network less able to propagate a full TNF signal to the cell (Fig. 6b). This may explain why we see reduced efficacy with TNF small molecules when TNFR1 signalling is driven by very high TNF concentrations, although it should be noted that these concentrations typically exceed those measured in patients with autoimmune disease such as rheumatoid arthritis^27^. In addition, the compounds described here are tool compounds with relatively low efficacy which can be attributed to their slow binding kinetics^20^.

**Fig. 7:**
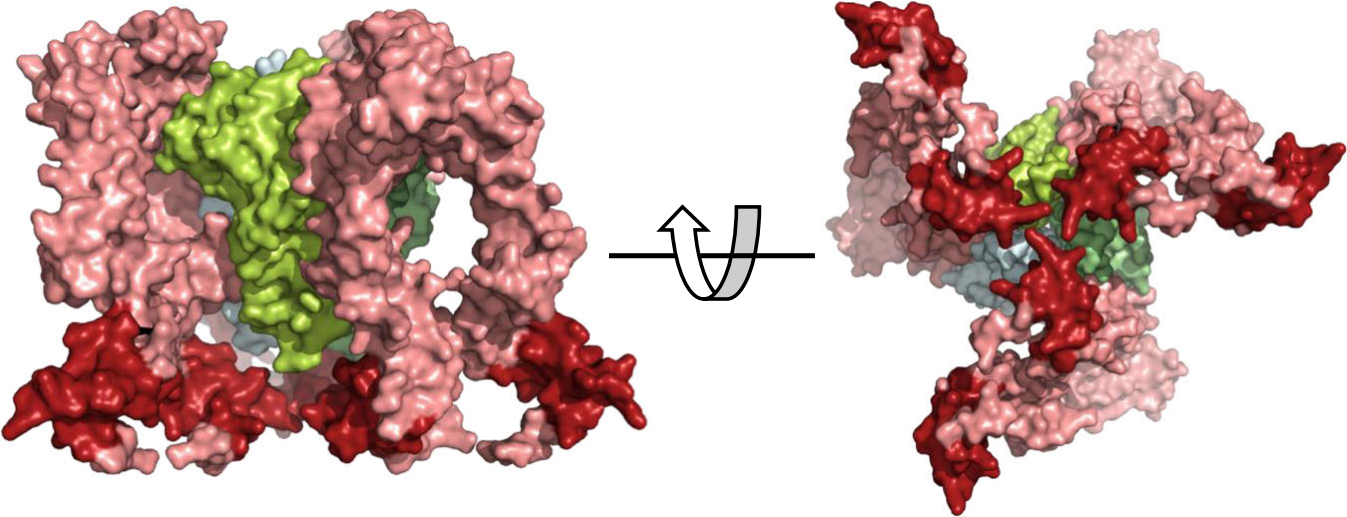
Model of TNF with long-form TNFR1 (41–201) dimers bound. Side and bottom view of surface rendered trimeric TNF (green) with three copies of long-form TNFR1 (41–201) dimers bound (pink). The fourth cysteine-rich domain of each receptor is highlighted in red.

Our data suggest that CRD4 has a critical role in the assembly of TNF–TNFR1 networks. In the PLAD dimer TNFR1 structure (1NCF [https://www.rcsb.org/structure/1NCF]) and the TNFβ–TNFR1 complex structure, CRD4 is mainly disordered^7,16^. The only TNFR1 structure where CRD4 is fully resolved is the antiparallel dimer structure 1EXT [https://www.rcsb.org/structure/1EXT]^28^. In order to visualise how the longer form of TNFR1 may engage TNF, we built a model of a long-form parallel receptor dimer by overlaying fully resolved receptor monomers from 1EXT [https://www.rcsb.org/structure/1EXT] onto the 1NCF [https://www.rcsb.org/structure/1NCF] receptor dimer. This long-form parallel dimer was then overlaid onto the three receptor-bound structure, resulting in a complex of TNF with three long-form receptor dimers bound (Fig. 7). There were no structural clashes within the long-form receptor dimer and only a minor clash between receptor and TNF suggesting that such a complex could form with only very minor changes in the receptor. In the overlay, the CRD4s within the receptor dimers were well separated (Fig. 7, highlighted in red at the bottom of each dimer pair), suggesting that a significant conformational change would be needed if they were to form any kind of direct contact, so it is probable that the long-form receptor dimer present in signalling networks has an alternative conformation to that shown.

The studies presented highlight important observations with regard to the interaction between TNF and TNFR1 and the assembly of larger networks, adding to our understanding of how TNF signals through TNFR1. The exact nature of the clustered TNF–TNFR1 complex in solution requires further characterisation but as it stands it offers a solution-based method for measuring the ability of compounds to disrupt what may represent the signalling network on cells. The data presented here, along with the tool compounds described, could be used to further elucidate the mechanism of TNF signalling. We could speculate that finding compounds that either completely block receptor binding at one site or block more than one receptor could inhibit signalling completely. Such small molecule inhibitors of TNF would have a significant impact on the breadth of the patient population able to access treatment for autoimmune diseases such as rheumatoid arthritis and Crohn’s disease.

## Methods

### Protein expression and purification

hTNF (residues 77–233) and mTNF (residues 82–235) were expressed with an N-terminal 6His-Smt3 tag under the P_BAD_ promoter in *Escherichia coli* TOP10 cells. Protein was purified by Ni^2+^-affinity chromatography (GE Healthcare) and SEC (Sephacryl S-100 HR, GE Healthcare). The His-Smt tag was removed with Ubiquitin-like-specific protease 1 (Ulp-1). For crystallography and analytical size exclusion chromatography hTNFR1 (residues 41–201, N54D, C182S) was expressed with an N-term ecdysteroid UDP-glucosyl transferase (EGT) signal peptide-8His-Tobacco etch virus (TEV) cleavage site-SNAP26b-thrombin cleavage site tag in *Trichoplusia ni* cells. Protein was purified by Ni^2+^-affinity chromatography (GE Healthcare) and SEC (Sephacryl S-100 HR, GE Healthcare). The His-TEV-SNAP-thrombin tag was removed with thrombin protease. For ion-mobility mass spectrometry and aggregation studies, hTNFR1(residues 41–201) and hTNFR1(residues 41–184, C182S) were expressed with a C-terminal TEV-human IgG1-Fc tag by transient transfection in CHOS-XE cells^29^ in the presence of kifunensine. Protein was captured on MabSelect SuRe resin (GE Healthcare) and hTNFR1 was released by on-column cleavage with TEV protease. The protein was subsequently deglycosylated with Endoglycosidase H (Endo-H) and further purified by SEC (Superdex 75, GE Healthcare). For SPR studies hTNFR1 (residues 41–201) were expressed with a C-terminal 6× lysine-TEV-human IgG1-Fc tag by transient transfection in CHOS-XE cells^29^. Protein was captured on MabSelect SuRe resin (GE Healthcare) and hTNFR1 with 6× lysine on the C terminus was released by on-column cleavage with TEV protease and further purified by SEC (Superdex 75, GE Healthcare).

### Ion-mobility mass spectrometry (IMS-MS)

Proteins (hTNF and hTNFR1) were desalted and buffer exchanged into 20 mM ammonium acetate, pH 7.4 prior to use using a combination of zeba spin columns (ThermoFisher, 7 kDa MWCO) followed by micro-dialysis (Thermo slide-a-lyzer mini dialysis units, 10 kDa MWCO). A stock of compound-bound hTNF was prepared by pre-incubating hTNF (20 µM, trimer) overnight at room temperature with a 10-fold molar excess of UCB-0595 (200 µM), 1% final DMSO concentration (an equivalent DMSO only stock was also prepared). The sample was shown to be 100% compound-bound by non-covalent time-of-flight mass spectrometry (Waters LCT Premier, equipped with Advion TriVersa NanoMate source). Samples of hTNF (±) UCB-0595 were incubated for 2 h at room temperature with an excess of hTNFR1 (hTNF:hTNFR1 = 5 µM:23 µM) and analysed by ion-mobility mass spectrometry (Waters Synapt G2 Q-TOF mass spectrometer, equipped with Advion TriVersa NanoMate source). For quantitative analysis of the receptor-bound state of hTNF, with and without compound, the receptor was added at a range of concentrations from 1 to 23 µM and analysed by ion-mobility mass spectrometry (Waters Synapt G2 Q-TOF mass spectrometer, equipped with Advion TriVersa NanoMate source). The following settings were utilised on the Waters Synapt G2 mass spectrometer: scan range 500–8000 *m*/*z* (Quad profile: 4000, 5000, 6000 (dwell: 30, ramp: 30)); Cone = 50 V; Source temp = 20 °C; Trap/transfer collision energy = off; Trap gas flow = 0.4 mL/min; Helium cell = 180 mL/min; IMS (N2) = 90 mL/min; Trap DC bias = 40 V; Mobility trapping manual release - not enabled; IMS wave delay = 450 μs; IMS wave velocity = 750 m/s; IMS wave height 40 V. Data were processed using Waters software MassLynx 4.1 and Drift Scope 2.0.

Quantitative analysis of IMS-MS data: Mass spectra data for each species were extracted within driftscope software and the resulting spectra were smoothed (50/5) and peak heights summed over all charge states. The ion counts of peaks corresponding to hTNF (no receptor-bound), hTNF + 1R (one receptor-bound), hTNF + 2R (two receptors bound) and hTNF+3R (three receptors bound) were measured. Normalised ion counts were calculated as the fraction of ions of each species divided by the total amount of ions counted. These values were used as equivalent to the molar fraction of each species in equilibrium. In order to derive equilibrium constants, the system in equilibrium was represented by the transformations:

$${\mathrm{TNF}} + {\mathrm{R}}\mathop{\longleftrightarrow}\limits^{{{\mathrm{K1}}}}{\mathrm{TNF}} + {\mathrm{1R}}$$

$${\mathrm{TNF}} + {\mathrm{1R}} + {\mathrm{R}}\mathop{\longleftrightarrow}\limits^{{{\mathrm{K2}}}}{\mathrm{TNF}} + {\mathrm{2R}}$$

$${\mathrm{TNF}} + {\mathrm{2R}} + {\mathrm{R}}\mathop{\longleftrightarrow}\limits^{{{\mathrm{K3}}}}{\mathrm{TNF}} + {\mathrm{3R}}$$

To calculate the set of dissociation constants K1, K2 and K3 in best agreement with native mass spectrometry data, values of K1, K2, K3 that produce molar fractions of the species TNF, TNF + 1R, TNF + 2R and TNF + 3R closest to the measured molar fractions of those species were obtained by minimisation of the function:

1 $$\begin{array}{ccccc}\\ {\mathrm{Error}}\left( {{\mathrm{K1}},{\mathrm{K2}},{\mathrm{K3}}} \right) & \\ \\ & = \mathop {\sum}\limits_{T0,R_{\mathrm{min}}}^{T0,R_{\mathrm{max}}} {\left[ {\left( f_{{\mathrm{TNF}}_{{\mathrm{calc}}}} - f_{{\mathrm{TNF}}_{{\mathrm{obs}}}} \right)^2 + \left( f_{{\mathrm{TNF}} + 1R_{{\mathrm{calc}}}} - f_{{\mathrm{TNF}} + 1R_{{\mathrm{obs}}}} \right)^2} \right.} \\ \\ & \left. { + \left( f_{{\mathrm{TNF}} + 2R_{{\mathrm{calc}}}} - f_{{\mathrm{TNF}} + 2R_{{\mathrm{obs}}}} \right)^2 + \left( f_{{\mathrm{TNF}} + 3R_{{\mathrm{calc}}}} - f_{{\mathrm{TNF}} + 3R_{{\mathrm{obs}}}} \right)^2} \right]_{T0,R}^{1/2}\\ \end{array}$$

where T0 represents the initial amount of TNF and *R*_min_, *R*, *R*_max_ represent the initial concentration of Receptor *R* assayed starting from *R*_min_ and ending at *R*_max_. Fractions *f*_obs_ are the molar fractions of species TNF, TNF + 1 R, TNF + 2 R and TNF + 3 R observed in equilibrium (ex. *f*_TNFobs)_ by native mass spectrometry measurements. *f*_calc_ are the molar fractions for each species (ex. *f*_TNFcalc_) calculated by solving the equilibrium equations using the BioNetGen BNGL modelling tool^30^ and taking as input the values of T0, R0 and K1, K2 and K3. The Error function was minimised using the brute force minimisation utility implemented within the SciPy/NumPy framework (http://docs.scipy.org/doc/numpy/index.html). The entire data processing analysis was implemented in the Python programming language (https://www.python.org/) calling BioNetGen routines when necessary. Data were visualised by plotting on the “Y” axis the molar fractions of all species in equilibrium and on the “X” axis, the concentration of receptor added to a fixed initial concentration of TNF. Symbols represent the observed molar fractions of species measured in the native mass spectrometry experiment and traces correspond to the expected concentrations calculated from the equilibrium constants K1, K2 and K3 (Fig. 1b).

### Surface plasmon resonance (SPR)

BIA (Biomolecular Interaction Analysis) uses SPR to detect the binding of TNFR in solution to TNF ± compound UCB-0595 complex pre-bound to TNFR immobilised on a CM5 sensor surface. HumanTNFR1 with a C-terminal 6× lysine tag was immobilised via amine coupling to approximately 750 RU at pH 5.5 in 10 mM sodium acetate on a Biacore T200 (GE Healthcare). Human TNF at 20 nM was pre-incubated for greater than 5 h ± UCB-0595 at 10 µM then flowed over the immobilised receptor in HBS-P buffer (10 mM HEPES pH 7.4, 0.15 M NaCl, 0.005% Surfactant P20) with 1% DMSO without dissociation. HumanTNFR1 monomer was then passed over in multicycles of: 6.25, 12.5, 25, 50, 100 and 200 nM. The surface was regenerated with 2 cycles of 10 mM HCl. The data were analysed in Biaevaluation software then presented in Graphpad Prism 7.

### Crystallisation and structure determination

The mTNF–hTNFR1 complex with UCB-4433 bound was formed by pre-incubating mTNF with a 6-fold molar excess of UCB-4433 then mixing with a 1:1 molar ratio of hTNFR1 (TNF MW based on the monomer) before isolating the complex using SEC (Superdex-200, GE Healthcare) in 10 mM HEPES pH 7.5, 150 mM NaCl and concentrating to 18.5 mg/mL. The complex crystallised by hanging drop vapour diffusion in 800 mM sodium potassium tartrate, 0.5% PEG5000 MME, 100 mM Tris pH 8.5. The apo form of the complex was solved following a similar procedure with an alternative compound that was absent from the final structure. In this case, the protein was crystallised at 9.2 mg/mL in 2.0 M Ammonium sulfate, 0.1 M BisTris pH 5.5. The hTNF(UCB-4433) complex was formed by incubating hTNF (4–7 mg/mL in 10 mM HEPES pH 7.5, 150 mM NaCl) overnight at 4 °C with 1-2 molar excess compound UCB-4433. The complex was crystallised by hanging drop vapour diffusion by mixing 0.5 µL of the complex with 0.5 µL of 21.44% w/v PEG3350, and 0.1 M Tris pH 9.0. Diffraction data for the compound-bound mTNF complex, apo mTNF complex and the hTNF(UCB-4433) complex were collected at the Argonne Photon Source, beamline 21-ID-F, Advanced Light Source, beamline 5.0.1 and the Argonne Photon Source, beamline 21-ID-G, respectively. Each dataset was integrated into XDS and scaled using SCALA^31,32^. The compound-bound structures were solved by molecular replacement using Phaser^33^ with input models based upon previously solved and unpublished crystal structures of hTNF bound with an unpublished UCB compound and an uncomplexed hTNFR1. The apo structure was solved by molecular replacement using Phaser with input models based upon previously solved crystal structures of uncomplexed apo hTNF and hTNFR1. Iterative manual model building using Coot^34^ and Refmac^35^ continued until R and Rfree converged. Model quality was validated using Coot and MolProbity^36^. Structures were validated using Molprobity prior to deposition in the Protein Data Bank (PDB ID’s: 7KP7, 7KP8 and 7KP9)^37,38^. Statistics for each crystal structure are provided in Supplementary Table 3. Stereo images of portions of the electron density maps can be found in Supplementary Fig. 4. Structural presentations were generated by PyMol (DeLano Scientific)^39^.

### In vitro network assembly assay

Determining degree of aggregation using spectrophotometry: When investigating the effect of overall protein concentration and the ratio of hTNF:hTNFR1 with the long and short forms of receptor, hTNF was incubated with hTNFR1 at the indicated concentrations, ratios and times (Fig. 5) in 10 mM HEPES pH 7.5, 150 mM NaCl (final sample volume = 100 μL). The degree of aggregation was quantified by measuring the absorbance at 600 nm using a BioTek Synergy 2 plate reader (BioTek Instruments Inc.) set to read samples after a 2 min fast shake to ensure complete mixing. For experiments measuring the effect of compound (Fig. 6a), the experiment was performed as described above except hTNF was pre-incubated with a 10-fold molar excess (over trimer) of UCB-9260 for a minimum of 17 h prior to mixing with hTNFR1.

Determining the degree of aggregation by gel densitometry: This method was applied when measuring the effect of the compound on the receptor–receptor interaction in the network. hTNF was incubated with a 5-fold molar excess of UCB-9260 or DMSO (final DMSO concentration of 4.8%) for a minimum of 24 h prior to mixing with TNFR1 at a ratio of 1 trimer: 3.2 receptors. The fixed 1:3.2 ratio mixes were prepared at a range of protein concentrations from 50 to 330 μM (based on the concentration of TNFR1). A duplicate set of samples containing hTNF only (no receptor) at an equivalent range of concentrations to that in the receptor containing samples was also prepared. Following a 1 h incubation at room temperature, the precipitate was removed by centrifugation at 16,000×*g* for 5 min at room temperature in an Eppendorf microcentrifuge. Samples of the soluble fraction were run on NuPAGE BisTris gels (ThermoFisher), stained with a Colloidal Blue Staining Kit (ThermoFisher) and destained with water until a transparent background was observed. The degree of precipitation in each sample was determined by densitometric analysis of the hTNF protein band in the soluble fraction using an ImageQuant LAS-3000 (GE Healthcare). Using the amount of TNF in the control sample (no receptor) as a reference for each receptor containing the sample, the fraction of TNF that had aggregated was determined and plotted against the protein concentration (based on the TNFR1 concentration) (Fig. 5f).

### NF-κβ reporter cell assay

Stimulation of HEK-Blue^TM^ cells by TNF leads to activation of the NF-κβ pathway. The HEK-Blue^TM^ CD40L SEAP (secreted embryonic alkaline phosphatase) reporter cell line used to determine TNF activity (Invivogen). Compound (UCB-9260) was titrated in DMSO and pre-incubated with either TNF or the anti-TNFR1 agonist antibody (R&D Systems, AF 225) for 1 h. Cells were added to the compound/stimulus mixture and further incubated for 18 h. SEAP was measured using the colorimetric substrate Quanti-blue TM (Invivogen). Percentage inhibitions for compound dilutions were calculated between a DMSO control and maximum inhibition (by excess anti-TNF biologic, or NF-κβ inhibitor—TPCA-1).

### Reporting summary

Further information on research design is available in the Nature Research Reporting Summary linked to this article.

## Supplementary information

Supplementary Information

Peer Review File

Description of Additional Supplementary files

Supplementary Software

Reporting Summary

## Data Availability

The data supporting this study are available from the corresponding author upon reasonable request. The structural data have been deposited with the Protein Data Bank under accession codes 7KP8 for mTNF–hTNFR1-UCB-4433, 7KP7 for mTNF–hTNFR1, and 7KP9 for hTNF-UCB-4433. Compound characterisation details can be found in Supplementary Notes 1–4. NMR spectral data are available for UCB-9260, UCB-4433 and UCB-0595 (Supplementary Figs. 5, 6, 8, 9, 11 and 12). High-resolution mass spectrometry (HRMS) is also available for UCB-9260, UCB-4433 and UCB-0595 (Supplementary Figs. 7, 10 and 13). Source data are provided with this paper.

## Source data

Source data

## Code availability

The code used to enable the derivation of dissociation constants from the quantitative analysis of the IM-MS data and instructions on how to run the script is available as Supplementary Software 1.
