# Supplementary material for: Structural insights into the disruption of TNF-TNFR1 signalling by small molecules stabilising a distorted TNF
Source: Nat Commun. 2021 Jan 25;12:582. doi: 10.1038/s41467-020-20828-3 (PMC7835368; doi:10.1038/s41467-020-20828-3)
Supplement: Supplementary file 1 — Supplementary Information [file 41467_2020_20828_MOESM1_ESM.pdf]

Structural insights into the disruption of TNF-TNFR1 signalling by small molecules stabilising a distorted TNF

David McMillan*[1], Carlos Martinez-Fleites[1,2], John Porter[1], David Fox 3rd [3], Rachel Davis[1], Prashant Mori[1], Tom Ceska[1], Bruce Carrington[1], Alastair Lawson[1], Tim Bourne[1], James O'Connell[1]

[1]UCB Pharma, Slough SL1 3WE, UK. [2]GlaxoSmithKline, Stevenage, SG1 2NY, UK. [3]UCB Pharma, Bainbridge Island, WA 98110, USA.

*Corresponding author: david.mcmillan@ucb.com

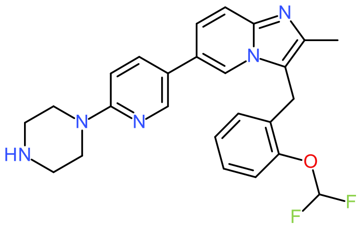

UCB-4433

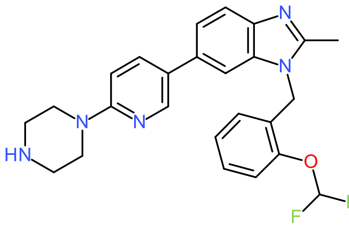

UCB-0595

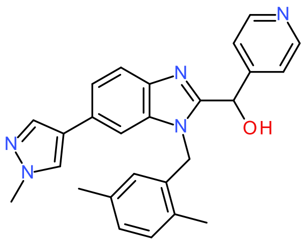

UCB-9260

Compound	RMSD (Å) relative to hTNF-UCB-5307 structure (PDB: 6OOZ)
**UCB-4433**	**0.33**
**UCB-0595**	**0.35**
**UCB-9260**	**0.31**

Supplementary
Figure 1. Structure of compounds used in this study and root-mean-square deviation (RMSD) values for each hTNF-compound structure: Using the hTNF-UCB-5307 crystal structure (PDB: 6OOZ [https://www.rcsb.org/structrure/6OOZ]) as a reference, RMSD values for the hTNF-compound structures of the other three compounds were calculated (table).

Compound	K1(nM)	K2(nM)	K3(nM)
No compound	0.01	0.02	0.22
UCB-0595	0.04	0.19	9612
Compound A	0.01	0.02	24328
Compound B	0.04	0.02	16965

Supplementary
Table 1. Effect of compounds on TNF-TNFR1 interaction: Quantitative analysis of IMS-MS data generated using hTNF plus compound (10 fold excess) over a range of hTNFR1 concentrations. Equilibrium constants for each receptor binding event are shown. Source data are provided as a Source Data file.

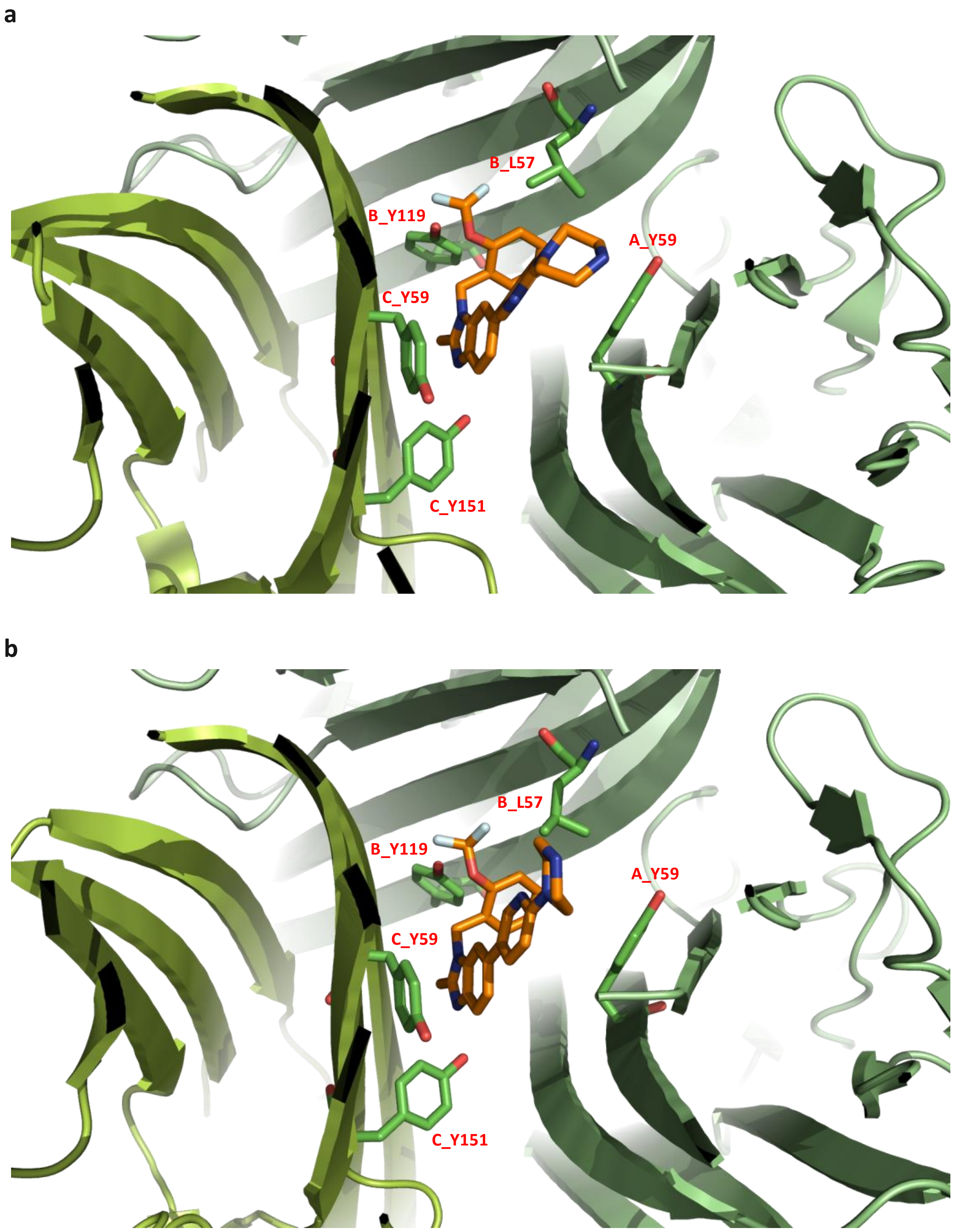

Supplementary
Figure 2: Crystal structure of human hTNF and mTNF with UCB-4433
Detail of UCB-4433 bound within the **(a)** hTNF (PDB code 7KP9 [https://www.rcsb.org/structrure/7KP9]) and **(b)** mTNF (PDB code 7KP8 [https://www.rcsb.org/structrure/7KP8]), with key residues involved in binding highlighted (sticks and labels). The mode of compound binding and overall structure of the human and mouse proteins is highly conserved (RMSD=0.5Å).
The soluble form of mouse TNF is 1 residue shorter than soluble human TNF. Based on structural alignments the missing residue is immediately after Pro 70. To maintain consistency in the numbering with human TNF (as used above) the structures of mTNF submitted to the PDB (7KP7 [https://www.rcsb.org/structrure/7KP7] and 7KP8 [https://www.rcsb.org/structrure/7KP8]) are numbered with a single digit shift after Pro70.

Residue	Movement Å
Y73	7.8
L75	7.1
T77	6.2
V91	7.3
I97	6.4

Supplementary
Table 2. Displacement of selected TNF residues involved in TNFR1 binding in the presence of compound: An overlay of apo and UCB-4433 bound mTNF (aligned through monomer C) was used to measure the displacement of monomer A. The degree of displacement (in Å) of selected residues on monomer A are shown (measurements taken on α–carbon atoms).

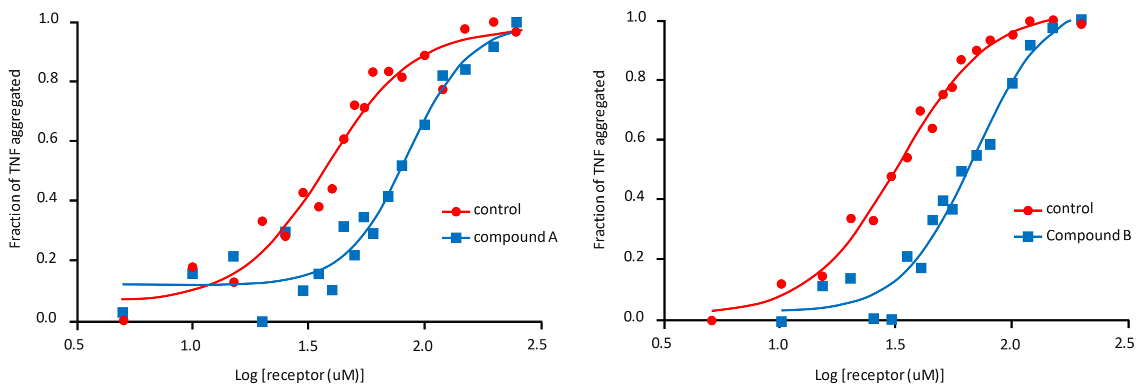

Supplementary
Figure 3. Inhibition of TNF-TNFR1 network assembly: Aggregation of hTNF +hTNFR1 at a fixed ratio of TNF:TNFR = 1:3.2 over a range of total protein concentrations with compounds A and B (5 fold excess over TNF) (blue trace) (left and right panels) and DMSO control (red trace). Source data are provided as a Source Data file.

Supplementary
Table 3 Crystallographic parameters

	**mTNF-hTNFR1-UCB-4433 PDB code: 7KP8**	**mTNF-hTNFR1 PDB code: 7KP7**	**hTNF-UCB-4433 PDB code: 7KP9**
**Data Collection**			
Space Group	$P\,4_1\,2_1\,2$	$P6_1$	$P2_1\,2_1\,2_1$
Cell dimensions			
a, b, c (Å)	133.58, 133.58, 141.45	139.22, 139.22, 138.34	54.43, 81.96, 93.24
α, β, γ (°)	90, 90, 90	90, 90, 120	90, 90, 90
Resolution (Å)	3.15 (3.32 - 3.15)	2.65 (2.72 - 2.65)	2.15 (2.21-2.15)
R_{meas}	0.19 (0.9)	0.07 (0.55)	0.01 (0.57)
I / σ(I)	8.3 (1.8)	19.98 (2.83)	14.19 (3.12)
Completeness (%)	99.9 (100)	99.5 (99.5)	99.9 (100)
Redundancy	4.9 (5.0)	3.7 (3.8)	4.9 (4.5)
**Refinement**			
Resolution (Å)	3.15	2.65	2.15
No. reflections	22,704	43,035	23.367
R_{work} / R_{free} overall	22.4 / 27.2	19.3 / 22.7	18.8/24.1
No. atoms			
Protein	4903	6500	3160
Ligands	33	225	33
Water	4	225	180
Average B-factor			
Protein	68	49	28
Ligand	61	99	22
Water	47	39	30
r.m.s. deviations			
Bond lengths (Å)	0.011	0.014	0.015
Bond angles (°)	1.22	1.43	1.49

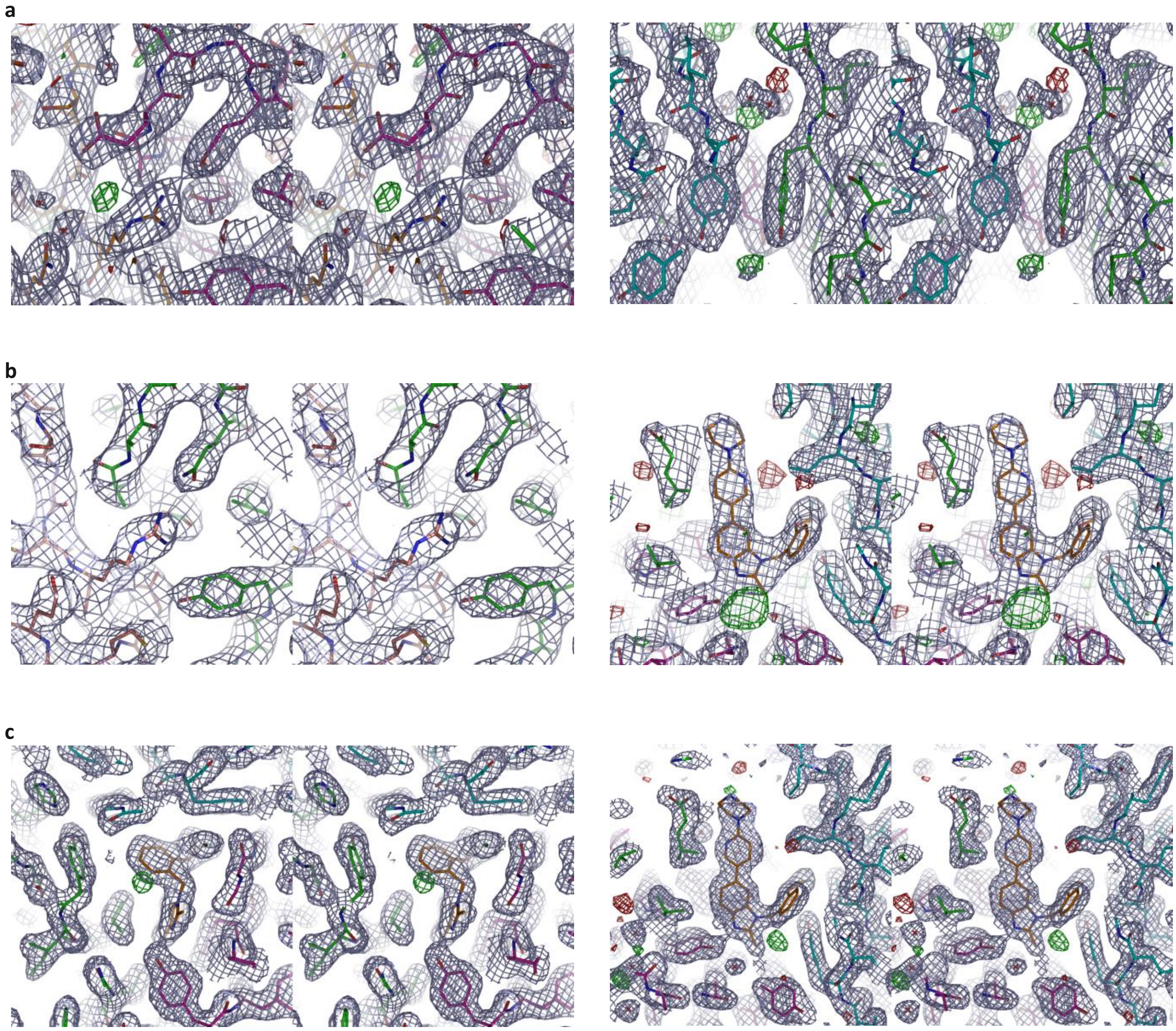

Supplementary
Figure 4: Electron density map of the crystal structures described in this manuscript
Stereo images showing electron density 2Fo-Fc maps contoured at 1.0σ (pale blue/grey) and Fo-Fc maps contoured at +/-3.0σ (green/red).
(a) mTNF-hTNFR1 (7KP7 [https://www.rcsb.org/structrure/7KP7]**):** Left panel - mTNF (magenta, chain C), hTNFR1 (orange, chain F). Central residue R77 of hTNFR1. Right panel - mTNF (green, chain A; cyan, chain B, magenta, chain C). Central residue is Y119 from chain A, B and C.
(b) mTNF-hTNFR1-UCB-4433 (7KP8 [https://www.rcsb.org/structrure/7KP8]**):** Left panel - mTNF (green, chain A; cyan, chain B; magenta, chain C), hTNFR1 (salmon, chain E). Central residue R77 of hTNFR1. Right panel- mTNF (green, chain A; cyan, chain B; magenta, chain C), UCB-4433 (orange)
(c) hTNF-UCB-4433 (7KP9 [https://www.rcsb.org/structrure/7KP9]**):** Left panel - mTNF (green, chain A; cyan, chain B; magenta, chain C), UCB-4433 (orange). Right panel - mTNF (green, chain A; cyan, chain B; magenta, chain C), UCB-4433 (orange).

Supplementary Note 1: Experimental and analytical details for synthetic analogues

Materials and reaction conditions

All solvents and reagents were used as received from commercial suppliers, unless noted otherwise. All reactions involving air- or moisture-sensitive reagents were performed under a nitrogen atmosphere using dried solvents and glassware.

Nomenclature

Compounds were named with the aid of ACD/Name Batch (Network) version 11.01 and/or Accelrys Draw 4.0 (IUPAC).

Abbreviations

DCM:	Dichloromethane
DMF:	N,N-Dimethylformamide
DMSO:	Dimethylsulfoxide
EtOAc:	Ethyl acetate
ES+:	Electrospray positive ionisation
h:	Hour
HPLC:	High performance liquid chromatography
LCMS:	Liquid Chromatography Mass Spectrometry
M:	Mass
MeOH:	Methanol
$MgSO_4$:	Magnesium sulfate
Na_2SO_4:	Sodium sulfate
r.t.:	Room temperature
RT:	Retention time
THF:	Tetrahydrofuran

Analysis by NMR

1H NMR spectra were recorded on a Bruker Avance III HD 500, 400 or 300 MHz spectrometer. The chemical shifts (δ) reported are given in parts per million (ppm) and the coupling constants (J) are in Hertz (Hz). The spin multiplicities are reported as s = singlet, d = doublet, t = triplet, q = quartet, dd = doublet of doublet, ddd = doublet of doublet of doublet, dt = doublet of triplet, td = triplet of doublet, and m = multiplet. Spectra were processed using MestReNova 10.0.

Analysis by LC-MS

LC-MS was performed on an Agilent 1200-6120 LC-MS system coupled to Detection (230 to 400 nm and 215 nm) and Mass Spec Detection Agilent 6120 Mass Spectrometer (ES) m/z 120 to 800 using *Method 1, 2 or 3*).

Automated preparative reverse phase HPLC

HPLC purification was performed on a Gilson system with a Gilson 306 pump, Gilson 215 autoinjector, Gilson 215 Fraction collector and a Gilson 156 UV detector (*Method 4*)

HPLC methods

Method 1:

Column: Waters X-Bridge, C18, 2.1 x 20 mm, 2.5 μm column.

Mobile phase A: 10 mM ammonium formate in water + 0.1% formic acid

Mobile phase B: acetonitrile + 5% mobile phase A + 0.1% formic acid

Gradient program (flow rate 1.0 mL/min, column temperature 40ºC):

Time	A%	B%
0.00	95.0	5.0
4.00	5.0	95.0
5.00	5.0	95.0
5.10	95.0	5.0

Method 2:

Column: Waters XSelect (C18, 30 x 2.1 mm, 3.5 μm) valve: 1

Flow Rate: 1 mL/minute

Column Temperature: 35ºC

Eluent A: 0.1% formic acid in acetonitrile

Eluent B: 0.1% formic acid in water

Lin. Gradient: t=0 min 5% A, t=1.6 min 98% A, t=3 min 98% A

Detection: DAD (220-320 nm)

Detection: MSD (ESI pos/neg) mass range: 100-800

Detection: ELSD (PL-ELS 2100): gasflow 1.2 mL/min, gas temp: 70ºC, neb: 50°C

Method 3:

Column: Waters XSelect (C18, 50 x 2.1 mm, 3.5 μm) valve: 2

Flow Rate: 0.8 mL/minute

Column Temperature: 35ºC

Eluent A: 0.1% formic acid in acetonitrile

Eluent B: 0.1% formic acid in water

Lin. Gradient: t=0 min 5% A, t=3.5 min 98% A, t=6 min 98% A

Detection: DAD (220-320 nm)

Detection: MSD (ESI pos/neg) mass range: 100-800

Detection: ELSD (PL-ELS 2100): gasflow 1.2 mL/min, gas temp: 70ºC, neb: 50ºC

Method 4:

Column: Luna C18, 21.2 mm, 5 mm column, pH 2.5.

Mobile phase A: 99.92% water and 0.08% formic acid.

Mobile phase B: 99.92% acetonitrile and 0.08% formic acid.

Gradient program (flow rate 25 mL/min, column temperature ambient): variable gradient.

Analysis by HRMS

All for all final compounds was performed on an Acquity UPLC - Xevo G2 MS.

Supplementary Note 2: Synthesis of UCB-9260

[1-(2,5-Dimethylbenzyl)-6-(1-methyl-1*H*-pyrazol-4-yl)-1*H*-benzimidazol-2-yl](pyridin-4-yl)methanol

5-Bromo-*N*-(2,5-dimethylbenzyl)-2-nitroaniline

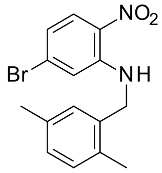

Sodium hydride (60% dispersion in oil, 0.82 g, 20.7 mmol) was added to a stirred solution of 5-bromo-2-nitroaniline (5.0 g, 23.0 mmol) in DMF (50 mL) at 0ºC. 2,5-Dimethyl-benzyl bromide (4.56 g, 23.0 mmol) was added and the reaction mixture was warmed to r.t. and stirred for 5 h. The reaction mixture was quenched with saturated aqueous ammonium chloride solution, extracted with ethyl acetate (3 x 50 mL), washed with water (2 x 30 mL), dried over anhydrous sodium sulfate and concentrated *in vacuo*. The residue was purified by column chromatography (SiO_2, 5% EtOAc/isohexane), yielding 5-bromo-*N*-(2,5-dimethylbenzyl)-2-nitroaniline (4.89 g, 63%) as a yellow solid. δ_H (300 MHz, d_6-DMSO) 8.42 (br s, 1H), 8.01 (d, *J* 8.8 Hz, 1H), 7.12-6.86 (m, 4H), 6.85 (d, *J* 7.2, 1.6 Hz, 1H), 4.54 (d, *J* 5.6 Hz, 2H), 2.28 (s, 3H), 2.21 (s, 3H).

5-Bromo-N^1-(2,5-dimethylbenzyl)benzene-1,2-diamine

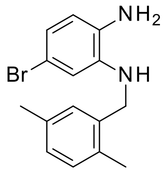

Tin (II) chloride (20.2 g, 89.4 mmol) was added to a stirred solution of 5-bromo-*N*-(2,5-dimethylbenzyl)-2-nitroaniline (10.0 g, 29.8 mmol) in EtOH (200 mL) and the reaction mixture was heated to 80ºC for 5 h. The reaction mixture was then concentrated *in vacuo* and the residue neutralized with saturated aqueous sodium bicarbonate solution and extracted with DCM (3 x 100 mL). The combined organics were washed with water (2 x 50 mL), extracted, dried over anhydrous sodium sulfate and concentrated *in vacuo*. The residue was purified by column chromatography (SiO_2, 5% MeOH/DCM), yielding 5-bromo-N^1-(2,5-dimethylbenzyl)benzene-1,2-diamine (5.4 g, 69%) as a dark brown oil. δ_H (300 MHz, d_6-DMSO) 7.08 (s, 1H), 7.06 (d, *J* 7.6 Hz, 2H), 6.97 (d, *J* 7.6 Hz, 1H), 6.53 (dd, *J* 8.4, 2.0 Hz, 1H), 6.47 (d, *J* 8.0 Hz, 1H), 6.45 (d, *J* 2.0 Hz, 1H), 5.06 (t, *J* 5.4 Hz, 1H), 4.77 (br s, 2H), 4.15 (d, *J* 5.2 Hz, 1H), 2.27 (s, 3H), 2.22 (s, 3H). LCMS (Method 1) (ES^+) (m/z) [M+H]+ 305/307 (Br pattern).

6-Bromo-1-(2,5-dimethylbenzyl)-1*H*-benzimidazole

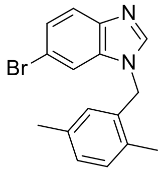

A mixture of 5-bromo-N^1-(2,5-dimethylbenzyl)benzene-1,2-diamine (0.40 g, 1.31 mmol) and formic acid (10 mL) was stirred at r.t. for 18 h. The reaction mixture was concentrated *in vacuo* and the residue partitioned between ethyl acetate and saturated aqueous sodium bicarbonate solution. The organic layer was dried over anhydrous sodium sulphate and concentrated *in vacuo*. The crude residue was purified by column chromatography (SiO_2, 20-75% EtOAc/ isohexane), yielding 6-bromo-1-(2,5-dimethylbenzyl)-1*H*-benzimidazole (0.20 g, 48%) as a white solid. δ_H (300 MHz, d_6-DMSO) 8.24 (s, 1H), 7.74 (d, *J* 1.7 Hz, 1H), 7.64 (d, *J* 8.6 Hz,

1H), 7.34 (dd, *J* 8.6, 1.9 Hz, 1H), 7.12 (d, *J* 7.7 Hz, 1H), 7.02 (d, *J* 7.8 Hz, 1H), 6.61 (s, 1H), 5.47 (s, 2H), 2.24 (s, 3H), 2.15 (s, 3H). LCMS (Method 1) (ES^+) (m/z) [M+H]+ 316/318 (Br pattern).

[6-Bromo-1-(2,5-dimethylbenzyl)-1*H*-benzimidazol-2-yl](pyridin-4-yl)methanol

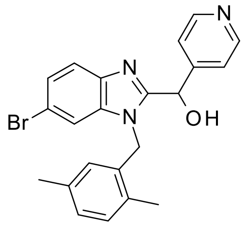

To diisopropylamine (2.8 mL) in THF (10 mL), cooled to 0ºC, was added *n*-butyllithium (12.5 mL, 1.6M in hexanes) and the resulting mixture was stirred at 0ºC for 10 minutes. An aliquot of this freshly prepared lithium diisopropylamide (1.8 mL, 1.62 mmol) was added to a solution of 6-bromo-1-(2,5-dimethylbenzyl)-1*H*-benzimidazole (0.25 g, 0.81 mmol) in THF (5 mL) at -78ºC. The reaction mixture was stirred for 2 h at -78ºC, then pyridine-4-carboxaldehyde (0.15 mL, 1.62 mmol) was added and the reaction mixture was stirred at -78ºC for 10 minutes. The mixture was quenched with saturated aqueous sodium chloride solution and allowed to warm to r.t. The mixture was extracted with ethyl acetate (3 x 40 mL). The organic layers were dried over anhydrous sodium sulphate and concentrated *in vacuo*. The residue was purified by column chromatography (SiO_2, 0-10% MeOH/DCM), yielding [6-bromo-1-(2,5-dimethylbenzyl)-1*H*-benzimidazol-2-yl](pyridin-4-yl)methanol (0.18 g, 51%) as a white solid. δ_H (400 MHz, d6-DMSO) 8.44 – 8.36 (m, 2H), 7.65 (d, *J* 8.6 Hz, 1H), 7.53 (d, *J* 2.0 Hz, 1H), 7.35 (dd, *J* 8.6, 1.8 Hz, 1H), 7.31 – 7.27 (m, 2H), 7.06 (d, *J* 7.7 Hz, 1H), 6.88 (d, 1H), 6.79 (d, *J* 5.5 Hz, 1H), 6.06 (d, *J* 5.4 Hz, 1H), 5.74 (s, 1H), 5.60 (d, *J* 17.3 Hz, 1H), 5.47 (d, *J* 17.3 Hz, 1H), 2.30 (s, 3H), 1.92 (s, 3H). LCMS (Method 1) (ES^+) (m/z) [M+H]+ 423/425 (Br pattern).

UCB-9260

[1-(2,5-Dimethylbenzyl)-6-(1-methyl-1*H*-pyrazol-4-yl)-1*H*-benzimidazol-2-yl](pyridin-4-yl)methanol

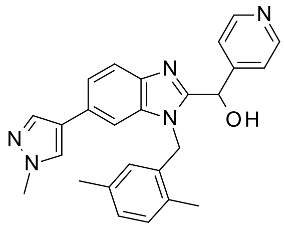

1-Methyl-4-(4,4,5,5-tetramethyl-[1,3,2]dioxaborolan-2-yl)-1*H*-pyrazole (0.064 g, 0.31 mmol), and a 2M aqueous solution of sodium carbonate (1 mL) were added to a solution of [6-bromo-1-(2,5-dimethylbenzyl)-1*H*-benzimidazol-2-yl](pyridin-4-yl)methanol (0.12 g, 0.29 mmol) in 1,4-dioxane:water (4:1, 5 mL) and the reaction mixture was degassed for 10 minutes. [1,1′-Bis(diphenylphosphino)ferrocene]dichloropalladium(II) (0.01 mg, 0.05 mmol) was added and the reaction mixture was degassed for 10 minutes, then heated to 100ºC for 60 minutes in a Biotage microwave reactor. Ethyl acetate was added and the mixture filtered through a Celite pad. The organic layer was separated, dried over anhydrous sodium sulphate, and concentrated *in vacuo*. The residue was purified by preparative HPLC (method 4), yielding [1-(2,5-dimethylbenzyl)-6-(1-methyl-1*H*-pyrazol-4-yl)-1*H*-benzimidazol-2-yl](pyridin-4-yl)methanol as a white solid (0.076 g, 62%). δ_H (400 MHz, d6-DMSO). 8.44 – 8.35 (m, 2H), 8.04 (s, 1H), 7.77 (d, *J* 0.8 Hz, 1H), 7.64 (d, *J* 8.3 Hz, 1H), 7.48 – 7.40 (m, 2H), 7.32 – 7.25 (m, 2H), 7.07 (d, *J* 7.6 Hz, 1H), 6.88 (d, *J* 7.7 Hz, 1H), 6.73 (broad s, 1H), 6.02 (s, 1H), 5.81 (s, 1H), 5.60 (d, *J* 17.3 Hz, 1H), 5.46 (d, *J* 17.3 Hz, 1H), 3.83 (s, 3H), 2.34 (s, 3H), 1.92 (s, 3H). ^{13}C NMR (101 MHz, DMSO) δ 154.96, 150.13, 149.58, 140.86, 136.95, 136.40, 135.03, 134.93, 131.93, 130.35, 128.11, 128.06, 127.80, 125.39, 122.95, 121.60, 120.36, 120.16, 106.78, 68.17, 44.97, 39.06, 21.09, 18.80. LCMS (Method 1) (ES^+) (m/z) [M+H]+ 424. HRMS (m/z): [M+H]+ calculated for $C_{26}H_{26}N_5O$, 424.2137; found, 424.2139.

Supplementary Note 3: Synthesis of UCB-4433

1-[2-(Difluoromethoxy)benzyl]-2-methyl-6-[6-(piperazin-1-yl)pyridin-3-yl]-1*H*-benzimidazole

6-Bromo-1-(2-difluoromethoxybenzyl)-2-methyl-1*H*-benzimidazole

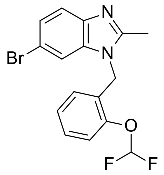

Step 1: To a solution of 2-(difluoromethoxy)benzylamine (5.00 g, 28.9 mmol) in DMF (50 mL) were added 4-bromo-2-fluoronitrobenzene (6.68 g, 30.3 mmol) and potassium carbonate (4.80 g, 34.68 mmol). The mixture was stirred at 100°C overnight. After this time the mixture was diluted with water (100 mL) and cooled to room temperature. The resultant solid precipitate was filtered off, washed with water and dried under vacuum, to give 5-bromo-*N*-[2-(difluoromethoxy)benzyl]-2-nitroaniline (10.00 g, 93%) as a yellow solid. δ_H (400 MHz, d_6-DMSO) 8.64 (t, *J* 6.1 Hz, 1H), 8.02 (d, *J* 9.1 Hz, 1H), 7.35-7.41 (m, 2H), 7.32 (t, $J_{H,F}$ 74.0 Hz, 1H), 7.21-7.28 (m, 2H), 7.09 (d, *J* 1.8 Hz,1H), 6.86 (dd, *J* 9.1, 1.9 Hz, 1H), 4.66 (d, *J* 6.2 Hz, 2H).

Step 2: To a solution of the foregoing material (3.00 g, 8.04 mmol) in ethanol (30 mL) and 10% HCl (15 mL) was added tin(II) chloride (4.57 g, 24.12 mmol). The mixture was stirred at 80°C overnight. After this time the mixture was basified with 10% aqueous NaOH solution (25 mL) and the mixture was cooled to room temperature. The mixture was then extracted with EtOAc (4 x 100 mL), and the combined organic layers were dried (Na_2SO_4), filtered and concentrated *in vacuo.* The crude product was purified by chromatography (SiO_2; 5-30% EtOAc/hexane), yielding 5-bromo-N^1-[2-(difluoromethoxy)benzyl]benzene-1,2-diamine (1.74 g, 63%) as a yellow oil. δ_H (400 MHz, d_6-DMSO) 7.32-7.39 (m, 2H), 7.27 (t, $J_{H,F}$ 74.2 Hz, 1H), 7.20-7.25 (m, 2H), 6.48-6.56 (m, 2H), 6.34 (d, *J* 2.0 Hz, 1H), 5.33 (t, *J* 5.8 Hz, 1H), 4.76 (s, 2H), 4.30 (d, *J* 5.8 Hz, 2H). LCMS (ES^+) (Method 1) (m/z) [M+H]+ 344.

Step 3: A solution of the foregoing material (545 mg, 1.59 mmol) in acetic acid (10 mL) was heated at 80 °C for 18 h. The reaction mixture was allowed to cool to ambient temperature, the volatiles were removed *in vacuo*, and the crude product was purified by chromatography (SiO_2; 20-60% EtOAc/hexane), yielding 6-bromo-1-(2-difluoromethoxybenzyl)-2-methyl-1*H*-

benzimidazole (479 mg, 82%) as a brown solid. δ_H (400 MHz, d_6-DMSO) 7.68 (d, *J* 1.8 Hz, 1H), 7.52 (d, *J* 8.7 Hz, 1H), 7.37-7.47 (m, 1H), 7.32 (t, *J* 74.2 Hz, 1H), 7.25-7.33 (m, 2H), 7.17 (td, *J* 7.68, 0.9 Hz, 1H), 6.75 (dd, *J* 7.6, 1.2 Hz, 1H), 5.49 (s, 2H), 2.48 (s, 3H). LCMS (ES^+) (Method 1) (m/z) [M+H]+ 368.

tert-Butyl 4-[5-[3-[[2-(difluoromethoxy)phenyl]methyl]-2-methyl-benzimidazol-5-yl]-2-pyridyl]piperazine-1-carboxylate

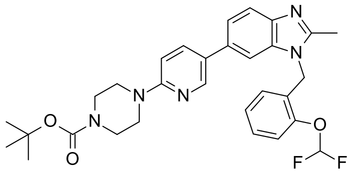

The following experiment was performed three times in parallel and the crude reaction mixtures combined for work-up and purification.

A mixture of *tert*-butyl 4-[5-(4,4,5,5-tetramethyl-1,3,2-dioxaborolan-2-yl)pyridin-2-yl]piperazin-1-ylcarboxylate (20 g, 54.5 mmol), 6-bromo-1-(2-difluoromethoxybenzyl)-2-methyl-1*H*-benzimidazole (24.5 g, 63.14 mmol), $PdCl_2$(dppf) (0.8 g, 1.1 mmol) and 2M aqueous sodium carbonate solution (25 mL) in 1,4-dioxane (200 mL) was degassed and heated under reflux in a nitrogen atmosphere overnight. The cooled reaction mixtures were combined, diluted with EtOAc and washed twice with brine. The organic layer was dried ($MgSO_4$) and the solvent was removed by rotary evaporation. The crude residue was purified by column chromatography (SiO_2; 60-100% EtOAc/hexane). The resulting material was triturated from ether, filtered, washed with more ether and dried to give *tert*-butyl 4-[5-[3-[[2-(difluoromethoxy)phenyl]methyl]-2-methyl-benzimidazol-5-yl]-2-pyridyl]piperazine-1-carboxylate (50.5 g, 56%) as a cream solid. δ_H (400MHz, d_6-DMSO) 8.44 (d, *J* 2.3 Hz, 1H), 7.85 (dd, J_1 8.9 Hz, J_2 2.6 Hz, 1H), 7.66 (d, *J* 1.3 Hz, 1H), 7.60 (d, *J* 8.4 Hz, 1H), 7.41 (m, 2H), 7.35 (t, $J_{H,F}$ 76 Hz, 1H), 7.27 (m, 1H), 7.16 (m, 1H), 6.92 (m, 1H), 6.79 (m, 1H), 5.54 (s, 2H), 3.52 (m, 4H), 3.44 (m, 4H), 2.50 (s, 3H), 1.43 (s, 9H). LCMS (ES^+) (Method 1) (m/z) [M+H]+ 550.

UCB-4433

1-[2-(Difluoromethoxy)benzyl]-2-methyl-6-[6-(piperazin-1-yl)pyridin-3-yl]-1*H*-benzimidazole

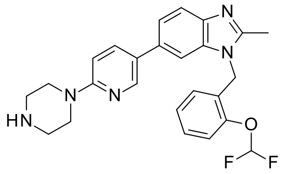

The following experiment was carried out twice in parallel.

Trifluoroacetic acid (100 mL) was added in portions to a chilled (0°C) solution of *tert*-butyl 4-[5-[3-[[2-(difluoromethoxy)phenyl]methyl]-2-methyl-benzimidazol-5-yl]-2-pyridyl]piperazine-1-carboxylate (25.25 g, 46 mmol) in DCM (50 mL) and stirred in an ice-bath for 30 minutes. Each reaction mixture was evaporated to a thin syrup, poured into a stirred mixture of $NaHCO_3$/ice/water and stirred for 1 h. The solid was filtered, washed several times with water and dried by suction. The solid was recrystallized from a 1:1 mixture of 2-propanol and 1-butanol to give 1-[2-(difluoromethoxy)benzyl]-2-methyl-6-[6-(piperazin-1-yl)pyridin-3-yl]-1*H*-benzimidazole (34 g, 82%) as a cream solid. δ_H (400MHz, d_6-DMSO) 8.41 (d, *J* 2.4 Hz, 1H), 7.81 (dd, J_1 8.9 Hz, J_2 2.6 Hz, 1H), 7.64 (d, *J* 1.3 Hz, 1H), 7.59 (m, 1H), 7.40 (m, 2H), 7.35 (t, $J_{H,F}$ 76 Hz, 1H), (m, 1H), 7.16 (m, 1H), 6.86 (d, *J* 8.9 Hz, 1H), 6.78 (m, 1H), 5.53 (s, 2H), 3.43 (m, 4H), 2.79 (m, 4H), 2.50 (s, 3H). LCMS (ES^+) (Method 1) (m/z) [M+H]+ 450.

UCB-4433 (as hydrochloride salt)

1-[2-(Difluoromethoxy)benzyl]-2-methyl-6-[6-(piperazin-1-yl)pyridin-3-yl]-1*H*-benzimidazole;hydrochloride

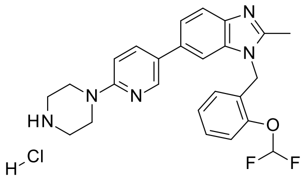

To 1-[2-(difluoromethoxy)benzyl]-2-methyl-6-[6-(piperazin-1-yl)pyridin-3-yl]-1*H*-benzimidazole (100 mg, 0.22 mmol) suspended in MeOH (4 mL) was added 2.25M HCl (0.10 mL, 0.22 mmol) and the flask swirled and left to stand at r.t. for 1h. The MeOH was evaporated, ethyl acetate (3 mL) was added and the resulting precipitate was filtered, washed with further

ethyl acetate and dried under vacuum to give 1-[2-(difluoromethoxy)benzyl]-2-methyl-6-[6-(piperazin-1-yl)pyridin-3-yl]-1*H*-benzimidazole;hydrochloride (90 mg, 84%) as a purple-grey solid. δ_H (400MHz, d_6-DMSO) 9.13 (s, 2H), 8.53 (d, *J* 2.5 Hz, 1H), 8.05 (d, *J* 1.4 Hz, 1H), 7.99 (dd, J_1 8.9 Hz, J_2 2.6 Hz, 1H), 7.93 – 7.76 (m, 2H), 7.46 (td, J_1 7.8 Hz, J_2 1.7 Hz, 1H), 7.37 (dd, J_1 7.8 Hz, J_2 1.7 Hz, 1H), 7.33 (t, $J_{H,F}$ 73.6 Hz, 1H), 7.28 (d, *J* 9.1 Hz, 1H), 7.24 (d, *J* 7.6 Hz, 1H), 7.08 (d, *J* 9.0 Hz, 1H), 5.79 (s, 2H), 3.81 (t, *J* 5.2 Hz, 4H), 3.19 (q, *J* 5.0 Hz, 4H), 2.83 (s, 3H). ^{13}C NMR (101 MHz, d_6-DMSO) δ 157.90, 152.95, 149.60, 146.01, 137.01, 135.39, 133.41, 130.78, 130.17, 125.81, 125.39, 124.92, 124.49, 119.38, 118.10, 117.35, 116.81, 115.20, 114.24, 110.01, 108.22, 44.24, 42.82, 42.23, 12.47. LCMS (ES^+) (Method 1) (m/z) [M+H]+ 450. HRMS (m/z): [M+H]+ calculated for $C_{25}H_{26}N_5OF_2$, 450.2100; found, 450.2106.

Supplementary Note 4: Synthesis of UCB-0595

3-[2-(Difluoromethoxy)benzyl]-2-methyl-6-[6-(piperazin-1-yl)pyridin-3-yl]imidazo[1,2-*a*]pyridine

N'-(5-Bromo-2-pyridyl)-*N*,*N*-dimethyl-acetamidine

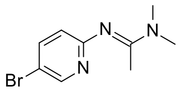

2-Amino-5-bromopyridine (10 g, 57.8 mmol) was suspended in methanol (100 mL) and *N*,*N*-dimethylacetamide dimethyl acetal (25.5 mL, 174.4 mmol) was added. The mixture was heated to reflux at 80ºC for 16 h. The mixture was concentrated under vacuum and ethyl acetate (80 mL) was added. The resulting material was washed with saturated aqueous sodium bicarbonate solution (50 mL) followed by water (3 x 50 mL) and then brine (50 mL). The organic layer was dried over sodium sulfate and concentrated under vacuum to afford *N'*-(5-bromo-2-pyridyl)-*N*,*N*-dimethyl-acetamidine (13.72 g, 98%) as a dark red oil. δ_H (500 MHz, $CDCl_3$) 8.34 (d, *J* 2.4 Hz, 1H), 7.62 (d, *J* 7.8 Hz, 1H), 6.69 (br s, 1H), 3.08 (s, 6H), 2.01 (s, 3H).

1-[2-(Difluoromethoxy)phenyl]ethan-1-one

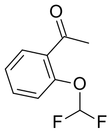

Potassium hydroxide (105 g, 1872 mmol) was suspended in a mixture of acetonitrile (200 mL) and water (200 mL) and cooled to approximately -20ºC. 1-(2-Hydroxyphenyl)ethanone (11.28 mL, 93.7 mmol) was added dropwise, followed by diethyl [bromo(difluoro)methyl]phosphonate (33.27 mL, 187.3 mmol) over 15 minutes. The mixture was then allowed to warm to room temperature over 1 hour. The mixture was extracted with ethyl acetate (3 x 200 mL), then the combined organic layers were washed with brine (50 mL), dried over magnesium sulfate and concentrated under vacuum. The mixture was purified by flash chromatography to afford 1-[2-(difluoromethoxy)phenyl]ethan-1-one (16.0 g, 92%) as a colourless oil. LCMS (ES^+) (Method 2) (m/z) [M+H]+ 187.

2-Bromo-1-[2-(difluoromethoxy)phenyl]ethan-1-one

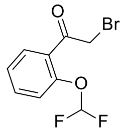

A solution of bromine (1.25 mL, 24.44 mmol) in glacial acetic acid (20 mL) was added dropwise over 60 minutes to a stirring solution of 1-[2-(difluoromethoxy)phenyl]ethan-1-one (4.6 g, 24.4 mmol) in glacial acetic acid (20 mL) in the dark. When the addition was complete the reaction was diluted with DCM (200 mL) and washed with water (200 mL). The aqueous layer was then extracted with DCM (50 mL). To the combined organic layers was added saturated aqueous sodium carbonate solution (100 mL), and further solid sodium carbonate was added portionwise with vigorous stirring until the mixture was neutralised. The organic phase was separated and the aqueous layer was extracted with DCM (2 x 50 mL). The combined organic layers were washed with brine (50 mL), dried over sodium sulphate, filtered and concentrated under vacuum to afford 2-bromo-1-[2-(difluoromethoxy)phenyl]ethan-1-one (6.48 g, 82%) as a light yellow oil. δ_H (500 MHz, $CDCl_3$) 7.83 (m, 1H), 7.58 (td, J_1 8.3 Hz, J_2 1.7 Hz, 1H), 7.34 (m, 1H), 7.20 (d, J 8.3 Hz, 1H), 6.64 (t, $J_{H,F}$ 72.9 Hz, 1H), 4.53 (s, 2H). LCMS (ES^+) (Method 3) (m/z) [M+H]+ 265/267 (Br pattern), 80% UV purity.

6-Bromo-3-{[2-(difluoromethoxy)phenyl]carbonyl}-2-methylimidazo[1,2-*a*]pyridine

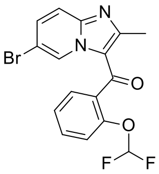

N'-(5-Bromo-2-pyridyl)-*N,N*-dimethyl-acetamidine (9.94 g, 41.1 mmol) and 2-bromo-1-[2-(difluoromethoxy)phenyl]ethan-1-one (10.9 g, 41.1 mmol) were combined in toluene (120 mL) and heated at 140°C for 10 minutes. The mixture was then allowed to cool gradually in the heating block for 1 h, before being cooled to room temperature. The volatiles were removed under vacuum and the residue was taken up in ethyl acetate (300 mL) and methanol (30 mL). The organic phase was washed with saturated aqueous sodium bicarbonate solution (150 mL) and the organic layer was dried over sodium sulphate, filtered and concentrated under vacuum to afford a red oil (~15 g). The residue was purified by flash chromatography, eluting with a

gradient of 0-100% ethyl acetate in heptane, to afford 6-bromo-3-{[2-(difluoromethoxy)phenyl]carbonyl}-2-methylimidazo[1,2-*a*]pyridine (9.94 g, 63.5%) as a pink solid. δ_H (500 MHz, $CDCl_3$) 9.96 (s, 1H), 7.58 (m, 3H), 7.38 (m, 3H), 6.52 (t, $J_{H,F}$ 73.5 Hz, 1H), 2.03 (s, 3H).

(6-Bromo-2-methylimidazo[1,2-*a*]pyridin-3-yl)[2-(difluoromethoxy)phenyl]methanol

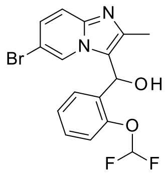

6-Bromo-3-{[2-(difluoromethoxy)phenyl]carbonyl}-2-methylimidazo[1,2-*a*]pyridine (9.94 g, 26.1 mmol) was suspended in methanol (200 mL). The mixture was then cooled to 0ºC in an ice bath and sodium borohydride (1.03 g, 27.4 mmol) was added. After 10 minutes the mixture was warmed to room temperature and stirred for 1 hour, after which time a light-coloured precipitate had formed. The mixture was reduced in volume *in vacuo* by approximately two-thirds and then diluted with ethyl acetate (400 mL). The organic phase was washed with saturated aqueous sodium bicarbonate solution (200 mL), dried over sodium sulphate and filtered, then concentrated *in vacuo*, to afford (6-bromo-2-methylimidazo[1,2-*a*]pyridin-3-yl)[2-(difluoromethoxy)phenyl]methanol (9.8 g, 98%) as a cream-coloured solid. δ_H (500 MHz, CD_3OD) 8.54 (s, 1H), 7.94 (m, 1H), 7.39 (m, 4H), 7.12 (m, 1H), 6.54 (m, 2H), 2.29 (s, 3H).

6-Bromo-3-[2-(difluoromethoxy)benzyl]-2-methylimidazo[1,2-*a*]pyridine

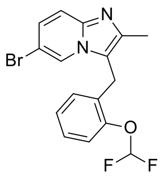

(6-Bromo-2-methylimidazo[1,2-*a*]pyridin-3-yl)[2-(difluoromethoxy)phenyl]methanol (9.6 g, 25.1 mmol) was suspended in DCM (200 mL). Boron trifluoride diethyl etherate (7.5 mL, 60.8 mmol) and triethylsilane (8 mL, 50.1 mmol) were added and the mixture was stirred at room

temperature for 6 h, before being left to stand at room temperature for 48 h. Further boron trifluoride diethyl etherate (3 mL, 24.3 mmol) and triethylsilane (2 mL, 12.5 mmol) were added and the mixture was stirred at room temperature for 6 h. The mixture was diluted with methanol (30 mL) to dissolve a small amount of precipitate, then the mixture was washed with saturated aqueous sodium bicarbonate solution (100 mL). The organic layer was dried over sodium sulfate and concentrated under vacuum to afford an orange gum. DCM (50 mL) was added, which caused a white precipitate to form. The precipitate was filtered and washed further with DCM (100 mL) and methanol (20 mL) to afford 6-bromo-3-[2-(difluoromethoxy)benzyl]-2-methylimidazo[1,2-*a*]pyridine (5.58 g, 54%) as a white solid. The filtrate was concentrated under vacuum and purified by flash chromatography, eluting with a gradient of 30-100% ethyl acetate in heptane, to afford a further quantity of 6-bromo-3-[2-(difluoromethoxy)benzyl]-2-methylimidazo[1,2-*a*]pyridine (1.18 g, 12%) as a pale orange solid. LCMS (ES^+) (Method 3) (m/z) [M+H]+ 367/369 (Br pattern), 90% UV purity.

tert-Butyl 4-(5-{3-[2-(difluoromethoxy)benzyl]-2-methylimidazo[1,2-*a*]pyridin-6-yl}pyridin-2-yl)piperazine-1-carboxylate

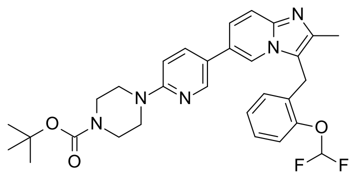

6-Bromo-3-[2-(difluoromethoxy)benzyl]-2-methylimidazo[1,2-*a*]pyridine (200 mg, 0.54 mmol) and *tert*-butyl 4-[5-(4,4,5,5-tetramethyl-1,3,2-dioxaborolan-2-yl)pyridin-2-yl]piperazine-1-carboxylate (316 mg, 0.81 mmol) were dissolved in 1,4-dioxane (20 mL) and a 2M aqueous solution of potassium carbonate (1 mL) was added. The mixture was flushed with nitrogen and bis[3-(diphenylphosphanyl)cyclopenta-2,4-dien-1-yl]iron-dichloropalladium-dichloromethane complex (12 mg, 0.01 mmol) was added. The mixture was heated at 90ºC under nitrogen for 16 h. Additional *tert*-butyl 4-[5-(4,4,5,5-tetramethyl-1,3,2dioxaborolan-2-yl)pyridin-2-yl]piperazine-1-carboxylate (150 mg, 0.39 mmol) and bis[3(diphenylphosphanyl)cyclopenta-2,4-dien-1-yl]iron-dichloropalladium-dichloromethane complex (12 mg, 0.01 mmol) were added and the mixture was heated at 90ºC under nitrogen for 4 h. The mixture was diluted with ethyl acetate (30 mL) and washed with water (2 x 10 mL), then brine (10 mL). The organic layer was dried over sodium sulphate, filtered and

concentrated under vacuum to yield a dark grey solid. The crude material was purified by flash chromatography, eluting with a gradient of 0-100% ethyl acetate in heptane, followed by 0-20% methanol in ethyl acetate. The resultant material was further purified by flash chromatography, eluting with a gradient of 0-5% methanol in DCM. The resultant material was then further purified by preparative HPLC (Method 4) to afford *tert*-butyl 4-(5-{3-[2-(difluoromethoxy)benzyl]-2-methylimidazo[1,2-*a*]pyridin-6-yl}pyridin-2-yl)piperazine-1-carboxylate (66 mg, 22%) as an off-white solid. δ_H (250 MHz, CD_3OD) 8.35-8.17 (m, 2H), 7.81-7.58 (m, 3H), 7.42-6.59 (m, 6H), 4.42 (s, 2H), 3.55 (br s, 8H), 2.48 (br s, 3H), 1.49 (s, 9H).

UCB-0595

3-[2-(Difluoromethoxy)benzyl]-2-methyl-6-[6-(piperazin-1-yl)pyridin-3-yl]imidazo[1,2-*a*]pyridine

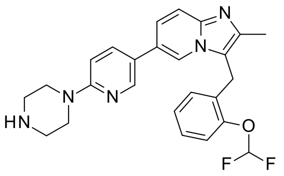

To *tert*-butyl 4-(5-{3-[2-(difluoromethoxy)benzyl]-2-methylimidazo[1,2-*a*]pyridin-6-yl}pyridin-2-yl)piperazine-1-carboxylate (318 mg, 0.58 mmol) were added 4M HCl in 1,4-dioxane (1 mL) and 1,4-dioxane (3 mL) and the mixture was stirred at room temperature for 5 h. The reaction mixture was diluted with ethyl acetate (30 mL) and washed with water (2 x 10 mL), then brine (10 mL). The organic layer was dried over sodium sulfate and concentrated under vacuum, then dried further in a vacuum oven, to afford 3-[2-(difluoromethoxy)benzyl]-2-methyl-6-[6-(piperazin-1-yl)pyridin-3-yl]imidazo[1,2-*a*]pyridine (58 mg, 93%) as an off-white solid. δ_H (500 MHz, CD_3OD) 8.89 (s, 1H), 8.45 (s, 1H), 8.39 (d, *J* 8.0 Hz, 1H), 8.23 (d, *J* 9.1 Hz, 1H), 8.00 (d, *J* 9.1 Hz, 1H), 7.57 (d, *J* 8.2 Hz, 1H), 7.43 (d, *J* 7.1 Hz, 1H), 7.36 (t, *J* 7.7 Hz, 1H), 7.22 (m, 2H), 6.95 (t, $J_{H,F}$ 73.9 Hz, 1H), 4.58 (s, 2H), 4.10 (s, 4H), 3.74-3.59 (m, 1H), 3.50 (s, 4H), 2.52 (s, 3H). δ_H (400MHz, d_6-DMSO) 8.39 (d, *J* 2.6 Hz, 1H), 8.30 (t, *J* 1.4 Hz, 1H), 7.81 (dd, J_1 8.9 Hz, J_2 2.7 Hz, 1H), 7.52 (dd, J_1 9.3 Hz, J_2 0.9 Hz, 1H), 7.50 – 7.41 (m, 1H), 7.36 – 7.24 (m, 2H), 7.25 – 7.19 (m, 1H), 7.18 – 7.10 (m, 2H), 7.04 (dd, J_1 7.7 Hz, J_2 1.8 Hz, 1H), 6.90 (d, *J* 8.9 Hz, 1H), 4.37 (s, 2H), 3.52 – 3.43 (m, 4H), 2.86 – 2.76 (m, 4H),

2.33 (s, 3H). ^{13}C NMR (101 MHz, DMSO) δ 158.98, 149.73, 145.79, 142.89, 141.32, 136.05, 130.28, 129.12, 128.65, 125.79, 123.13, 122.66, 122.03, 119.84, 119.67, 118.67, 118.20, 117.10, 116.65, 114.54, 107.37, 45.96, 45.63, 23.28, 13.84. LCMS (Method 1) (ES^+) (m/z) [M+H]+ 450, 94% UV purity. HRMS (m/z): [M+H]+ calculated for $C_{25}H_{26}N_5OF_2$, 450.2100; found, 450.2101.

Supplementary Fig 5: 1H NMR spectrum of UCB-9260

The spectrum was recorded on a Bruker Avance III HD 400mHz spectrometer

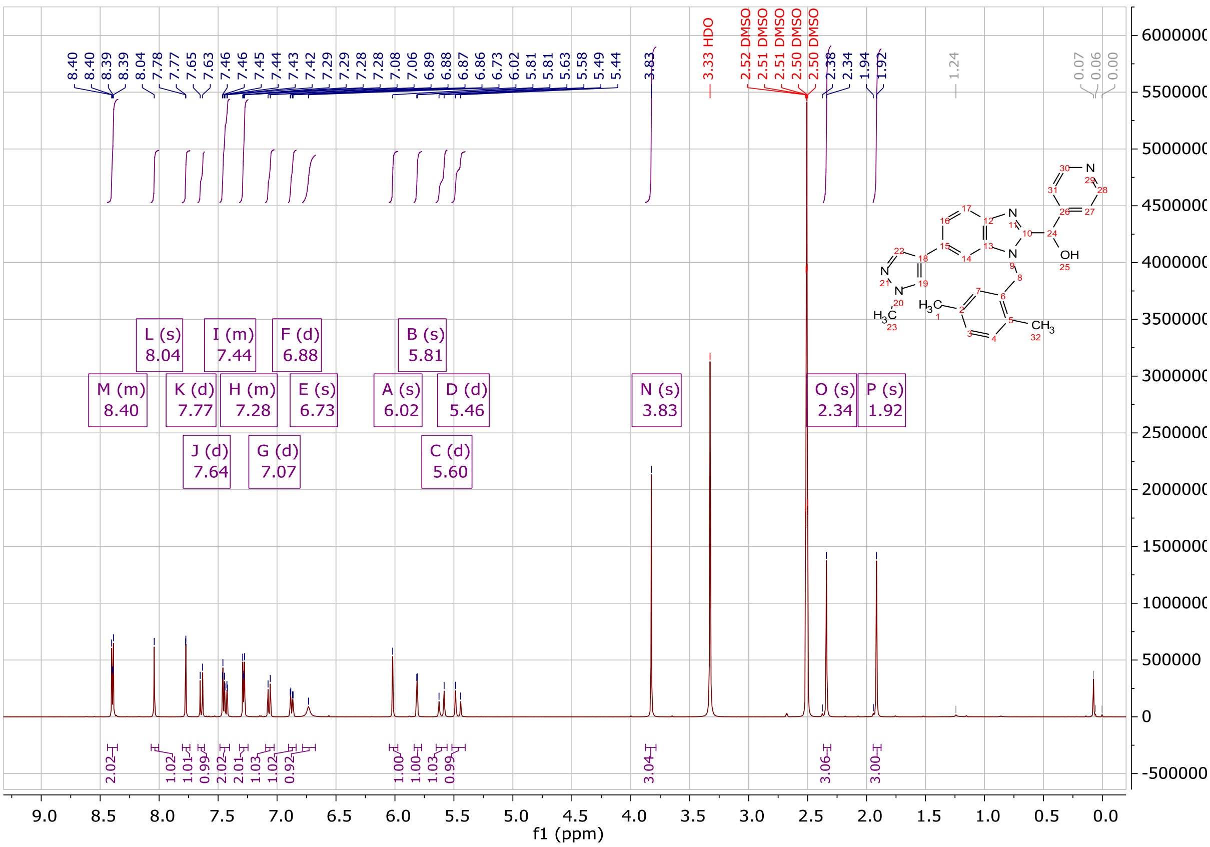

Supplementary Fig 6: 13C NMR spectrum of UCB-9260 (101 MHz)

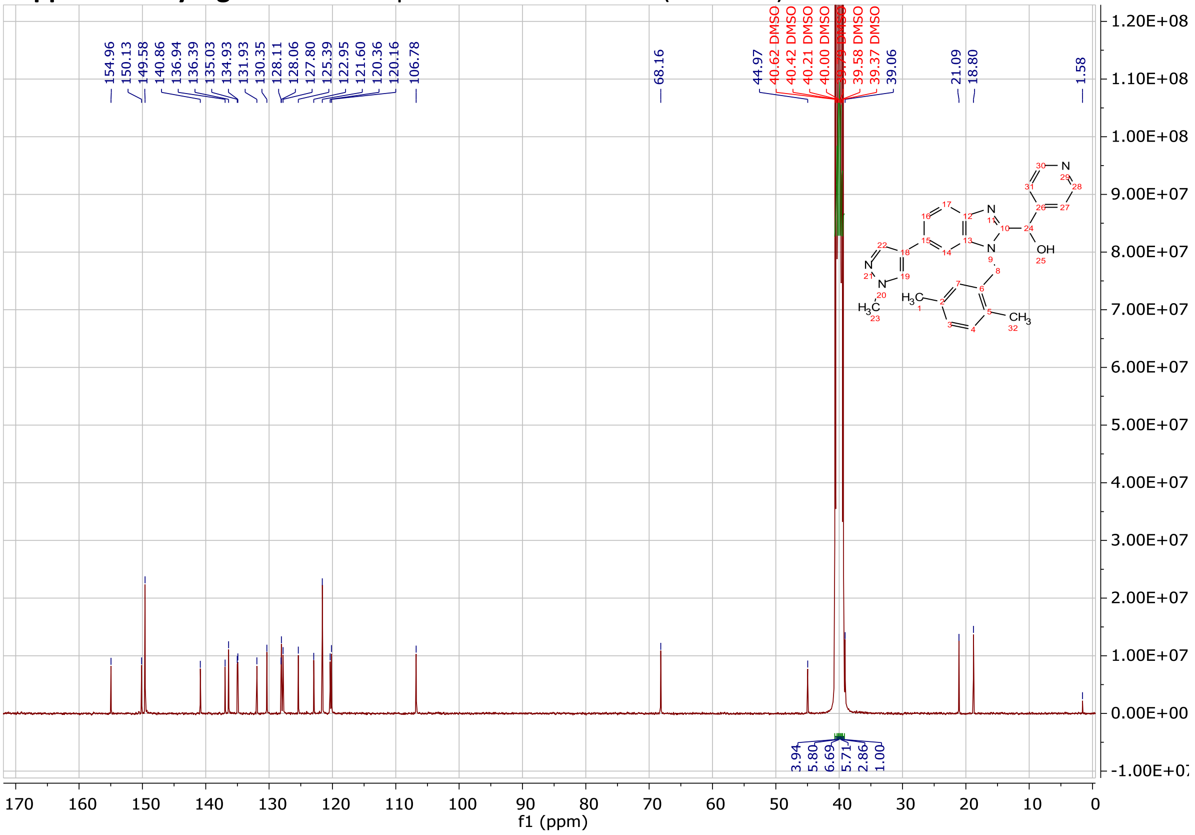

Supplementary Fig 7: HRMS spectrum of UCB-9260

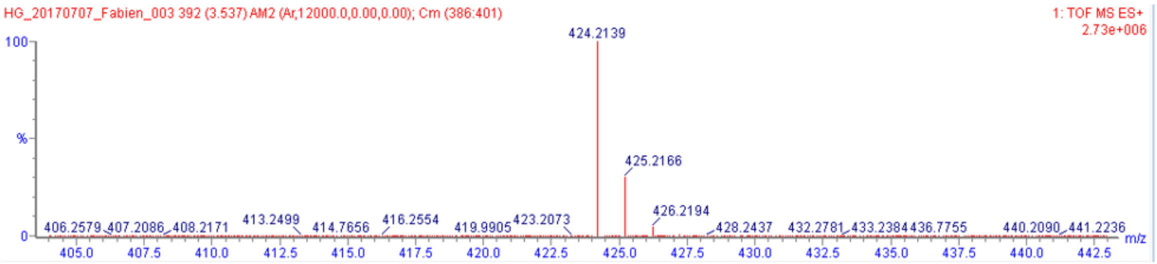

Supplementary Fig 8: 1H NMR spectrum of UCB-4433
The spectrum was recorded on a Bruker Avance III HD 400mHz spectrometer

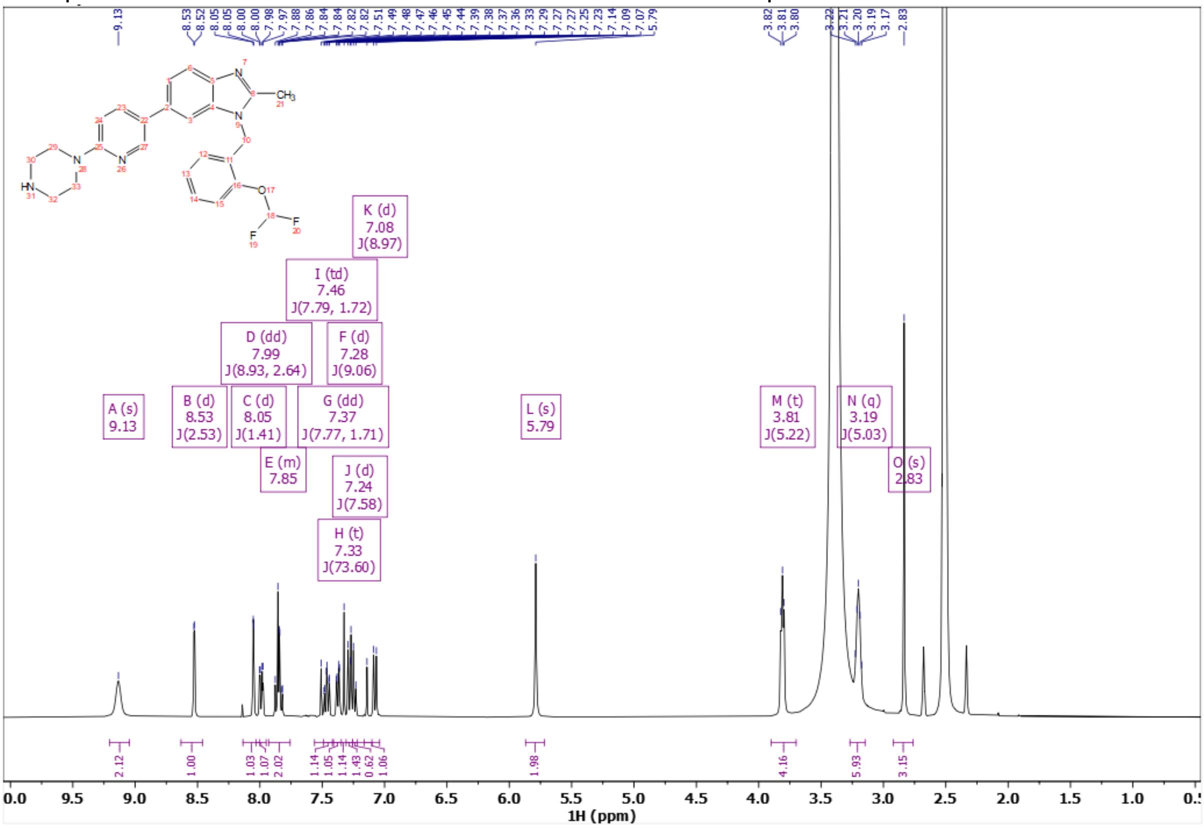

Supplementary Fig 9: 13C NMR spectrum of UCB-4433 (101 MHz)

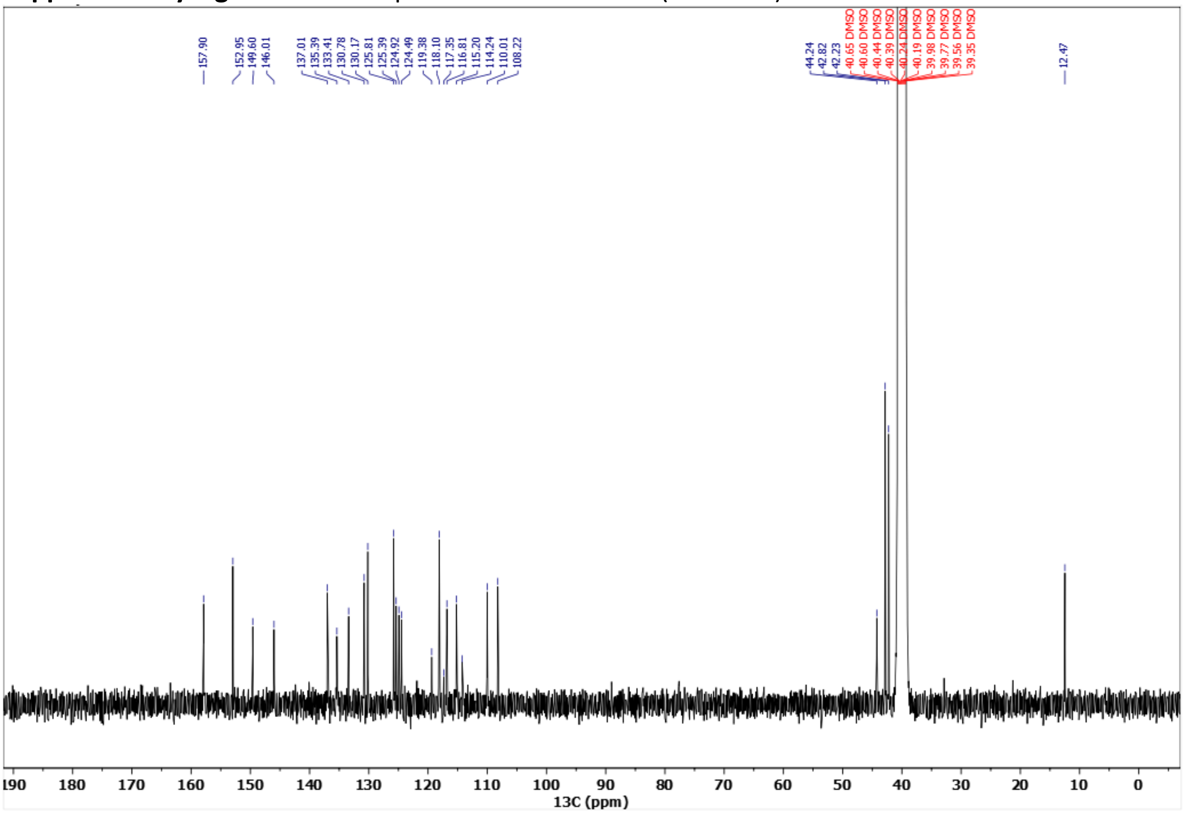

Supplementary Fig 10: HRMS spectrum of UCB-4433

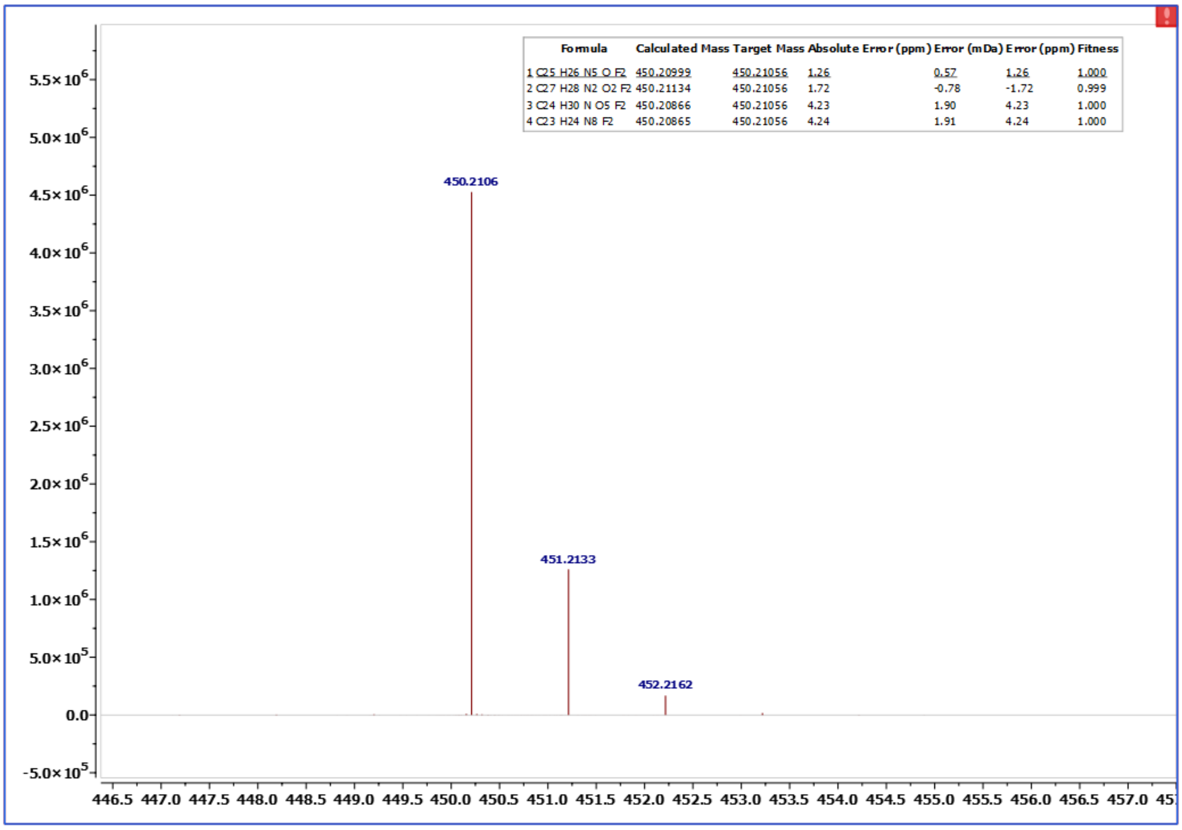

	Formula	Calculated Mass	Target Mass	Absolute Error (ppm)	Error (mDa)	Error (ppm)	Fitness
1	C25 H26 N5 O F2	450.20999	450.21056	1.26	0.57	1.26	1.000
2	C27 H28 N2 O2 F2	450.21134	450.21056	1.72	-0.78	-1.72	0.999
3	C24 H30 N O5 F2	450.20866	450.21056	4.23	1.90	4.23	1.000
4	C23 H24 N8 F2	450.20865	450.21056	4.24	1.91	4.24	1.000

Supplementary Fig 11: 1H NMR spectrum of UCB-0595

The spectrum was recorded on a Bruker Avance III HD 400mHz spectrometer

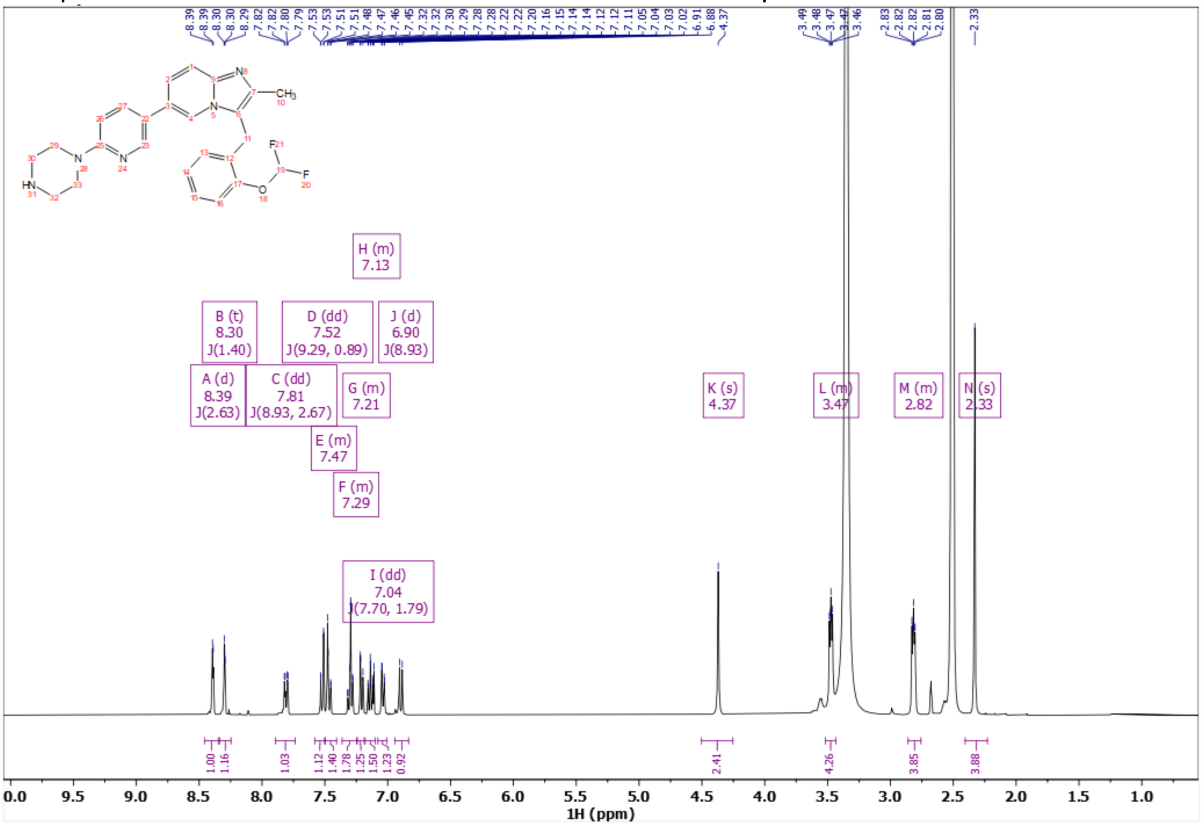

Supplementary Fig 12: 13C NMR spectrum of UCB-0595 (101 MHz)

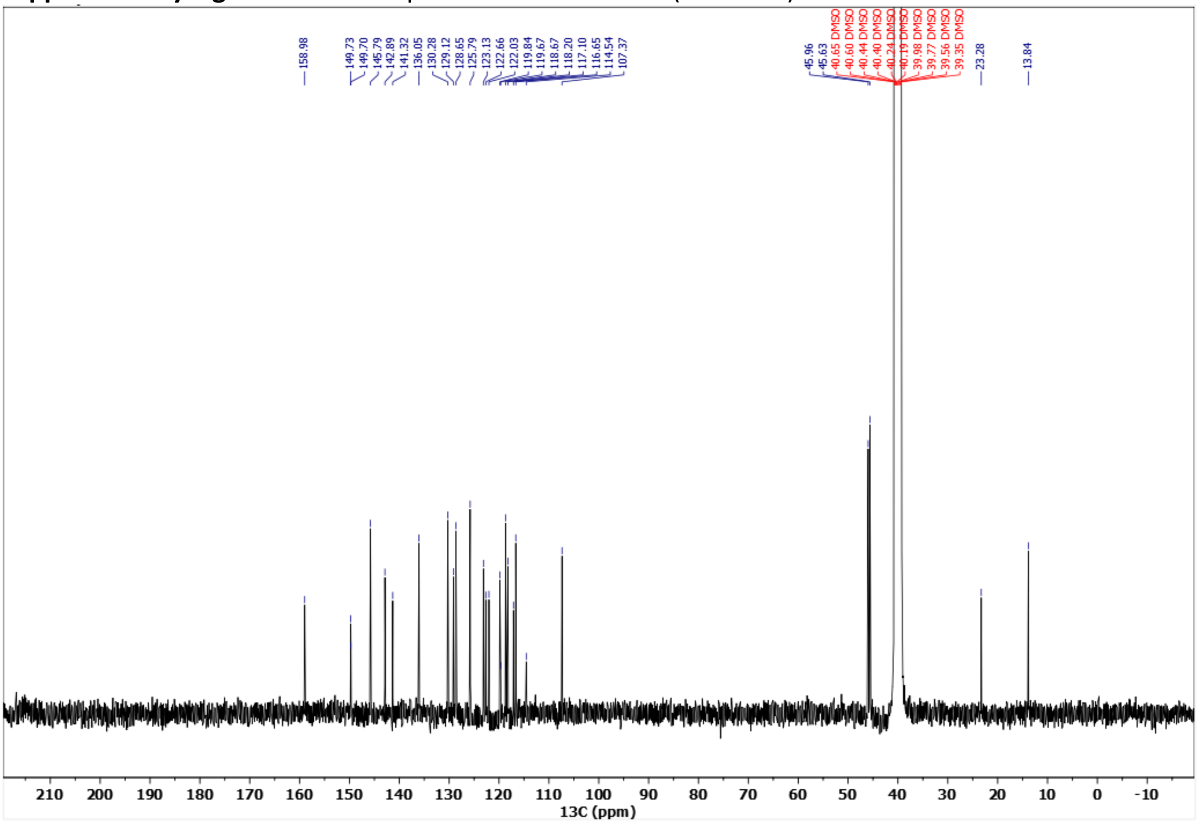

Supplementary Fig 13: HRMS spectrum of UCB-0595

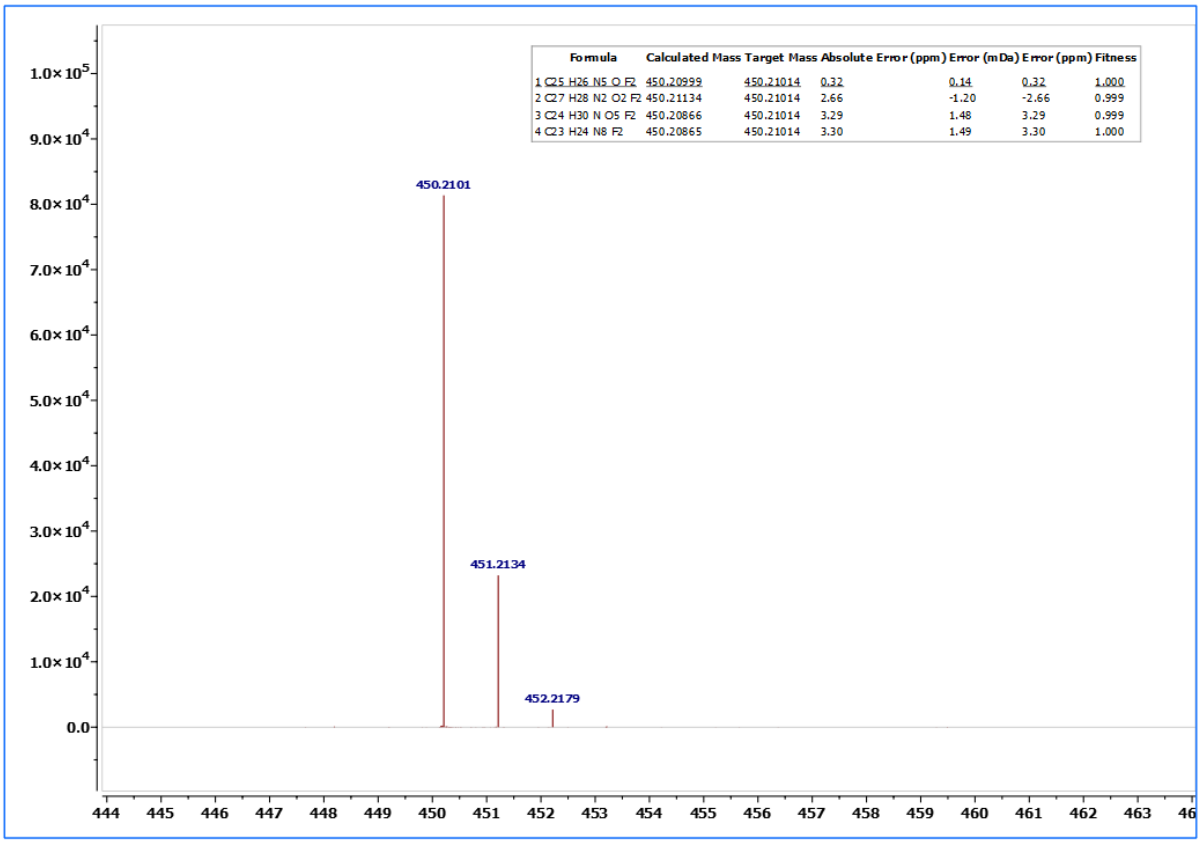

	Formula	Calculated Mass	Target Mass	Absolute Error (ppm)	Error (mDa)	Error (ppm)	Fitness
1	C25 H26 N5 O F2	450.20999	450.21014	0.32	0.14	0.32	1.000
2	C27 H28 N2 O2 F2	450.21134	450.21014	2.66	-1.20	-2.66	0.999
3	C24 H30 N O5 F2	450.20866	450.21014	3.29	1.48	3.29	0.999
4	C23 H24 N8 F2	450.20865	450.21014	3.30	1.49	3.30	1.000
