# Supplementary material for: Structural insights into the disruption of TNF-TNFR1 signalling by small molecules stabilising a distorted TNF
Source: Nat Commun. 2021 Jan 25;12:582. doi: 10.1038/s41467-020-20828-3 (PMC7835368; doi:10.1038/s41467-020-20828-3)
Supplement: Supplementary file 3 — Description of Additional Supplementary files [file 41467_2020_20828_MOESM3_ESM.docx]

**Description of Additional Supplementary Files**

**File Name**: Supplementary Software 1

**Description**: BioNetGen files, Python script files and a document detailing instructions on how to run the script for quantitative analysis of IMS-MS data.
