# Supplementary material for: Structural insights into the disruption of TNF-TNFR1 signalling by small molecules stabilising a distorted TNF
Source: Nat Commun. 2021 Jan 25;12:582. doi: 10.1038/s41467-020-20828-3 (PMC7835368; doi:10.1038/s41467-020-20828-3)
Supplement: Supplementary file 4 — Supplementary Software [file 41467_2020_20828_MOESM4_ESM.zip › Instructions on running the script.docx]

Instructions on running the script

In order to run the script, users will need to have a python 2.7 installation with the packages detailed in the python script (“massSpec_v4.py”) from line 4 to 20. In addition, script will need access to perl ([https://www.perl.org/](https://eur02.safelinks.protection.outlook.com/?url=https%3A%2F%2Fwww.perl.org%2F&data=02%7C01%7CDavid.McMillan%40ucb.com%7Cbd65696fd518449fc64f08d8348af231%7C237582ad3eab4d44868806ca9f2e613b%7C0%7C0%7C637317119687392267&sdata=khB27bVkwv%2BEl2x92IZaPFxuXYPIaB2rcW50EU6r33U%3D&reserved=0))  and BioNetGen perl file BNG.pl as specified in script line 301. The BNG.pl file can be obtained from [http://michaelsneddon.net/nfsim/download/](https://eur02.safelinks.protection.outlook.com/?url=http%3A%2F%2Fmichaelsneddon.net%2Fnfsim%2Fdownload%2F&data=02%7C01%7CDavid.McMillan%40ucb.com%7Cbd65696fd518449fc64f08d8348af231%7C237582ad3eab4d44868806ca9f2e613b%7C0%7C0%7C637317119687402257&sdata=XaNg9ej2Z0Z0nstTWVH5DAn1mAyFmtfkU27UeUzLswI%3D&reserved=0))

To run the script on a specific data, save in a folder the BioNetGen file (“version_test.bngl”) , the python script (“massSpec_v4.py”) and a file containing the data as molar fraction of all TNF / TNF receptor species in the columns and initial TNF receptor concentrations in the rows. Data file should be named 'Data.txt'. Finally, in a computer terminal type:

⮚  python massSpec_v4.py
