# Supplementary material for: Structural insights into the disruption of TNF-TNFR1 signalling by small molecules stabilising a distorted TNF
Source: Nat Commun. 2021 Jan 25;12:582. doi: 10.1038/s41467-020-20828-3 (PMC7835368; doi:10.1038/s41467-020-20828-3)
Supplement: Supplementary file 5 — Reporting Summary [file 41467_2020_20828_MOESM5_ESM.pdf]

nature research

Corresponding author(s): David McMillan

Last updated by author(s): Dec 8, 2020

Reporting Summary

Nature Research wishes to improve the reproducibility of the work that we publish. This form provides structure for consistency and transparency in reporting. For further information on Nature Research policies, see our Editorial Policies and the Editorial Policy Checklist.

Statistics

For all statistical analyses, confirm that the following items are present in the figure legend, table legend, main text, or Methods section.

n/a	Confirmed	
☐	☒	The exact sample size (n) for each experimental group/condition, given as a discrete number and unit of measurement
☐	☒	A statement on whether measurements were taken from distinct samples or whether the same sample was measured repeatedly
☒	☐	The statistical test(s) used AND whether they are one- or two-sided<br>*Only common tests should be described solely by name; describe more complex techniques in the Methods section.*
☒	☐	A description of all covariates tested
☒	☐	A description of any assumptions or corrections, such as tests of normality and adjustment for multiple comparisons
☒	☐	A full description of the statistical parameters including central tendency (e.g. means) or other basic estimates (e.g. regression coefficient) AND variation (e.g. standard deviation) or associated estimates of uncertainty (e.g. confidence intervals)
☒	☐	For null hypothesis testing, the test statistic (e.g. F, t, r) with confidence intervals, effect sizes, degrees of freedom and P value noted<br>*Give P values as exact values whenever suitable.*
☒	☐	For Bayesian analysis, information on the choice of priors and Markov chain Monte Carlo settings
☒	☐	For hierarchical and complex designs, identification of the appropriate level for tests and full reporting of outcomes
☒	☐	Estimates of effect sizes (e.g. Cohen's d, Pearson's r), indicating how they were calculated

Our web collection on statistics for biologists contains articles on many of the points above.

Software and code

Policy information about availability of computer code

Data collection	X-ray experiments experiments were performed on custom software for SSRL beamline 7-1 and ALS beamline 5.0.3

Data analysis	Mass Spectroscopy Software:<br>MassLynx 4.1<br>DriftScope 2.0<br><br>Quantitative analysis of IMS-MS data:<br>NumPy v1.8<br>Python v3.3<br><br>Biacore software:<br>Biacore T100 BIAevaluation software version 1.1<br>Graphpad Prism 7<br><br>Crystallography tools:<br>Data reduction - XDS (Versions January 30, 2009, December 31, 2001, and July 4, 2012)<br>Data scaling - XSCALE (Versions January 30, 2009, December 31, 2001, and July 4, 2012)<br>Refinement - CCP4 program suite, Refmac5 (Version 5.5.0109 and 5.6.0117)<br>Validation - Molprobity<br>Validation - wwPDB validation service

For manuscripts utilizing custom algorithms or software that are central to the research but not yet described in published literature, software must be made available to editors and reviewers. We strongly encourage code deposition in a community repository (e.g. GitHub). See the Nature Research guidelines for submitting code & software for further information.

Data

Policy information about availability of data

All manuscripts must include a data availability statement. This statement should provide the following information, where applicable:

- Accession codes, unique identifiers, or web links for publicly available datasets
- A list of figures that have associated raw data
- A description of any restrictions on data availability

The datasets generated during and/or analysed during the current study are available from the corresponding author on reasonable request.

Field-specific reporting

Please select the one below that is the best fit for your research. If you are not sure, read the appropriate sections before making your selection.

[x] Life sciences   [ ] Behavioural & social sciences   [ ] Ecological, evolutionary & environmental sciences

For a reference copy of the document with all sections, see nature.com/documents/nr-reporting-summary-flat.pdf

Life sciences study design

All studies must disclose on these points even when the disclosure is negative.

Sample size	No sample size calculation was made. Where n=1 data is used, data in the published experiment is supported by a number of experiments on compounds with the same mode of action.
Data exclusions	No data were excluded from the analysis
Replication	Replication with individual compounds for some experiments were not done due to the complex nature of the experiments, however multiple compounds from the same chemical series were tested and reproducibility was good. Where applicable this is highlighted in the manuscript. The reporter cell assay was replicated n=5 (100pM TNF) and n-8 (10pM TNF) and were successful.
Randomization	Randomization is not relevant to this study as there was no possibility of bias in the experiments performed.
Blinding	Blinding is not relevant to this study as the readouts for all experiments were not subjective.

Reporting for specific materials, systems and methods

We require information from authors about some types of materials, experimental systems and methods used in many studies. Here, indicate whether each material, system or method listed is relevant to your study. If you are not sure if a list item applies to your research, read the appropriate section before selecting a response.

Materials & experimental systems

n/a	Involved in the study	
☐	☒	Antibodies
☐	☒	Eukaryotic cell lines
☒	☐	Palaeontology and archaeology
☒	☐	Animals and other organisms
☒	☐	Human research participants
☒	☐	Clinical data
☒	☐	Dual use research of concern

Methods

n/a	Involved in the study	
☒	☐	ChIP-seq
☒	☐	Flow cytometry
☒	☐	MRI-based neuroimaging

Antibodies

Antibodies used	NF-kB reporter cell assay: anti-TNFR1 agonist antibody (R&D Systems AF225)
Validation	The antibody was confirmed to be applicable for this application as detailed in the data sheet provided by the commercial supplier

Eukaryotic cell lines

Policy information about cell lines

Cell line source(s)	CHOS-XE cells were developed within UCB.<br>Trichoplusia ni (T.ni) cells were sourced from ThermoFisher,<br>HEK-Blue CD40L cells were purchased from Invivogen (hkb-CD40)
Authentication	CHOS-EX and T.ni cells have been used to produce multiple proteins for multiple projects by UCB with expected results (expected glycosylation patterns and no unexpected post-translational modifications as determined by LCMS). HEK-Blue CD40L cells responded to TNFa ligand in-accordance with information from the supplier (signaling was blocked by an anti-TNFa neutralizing antibody).
Mycoplasma contamination	All cell lines tested negative for mycoplasma contamination
Commonly misidentified lines<br>(See ICLAC register)	no commonly misidentified cell lines were used
