# Supplementary material for: Structural insights into the disruption of TNF-TNFR1 signalling by small molecules stabilising a distorted TNF
Source: Nat Commun. 2021 Jan 25;12:582. doi: 10.1038/s41467-020-20828-3 (PMC7835368; doi:10.1038/s41467-020-20828-3)
Supplement: Supplementary file 2 — Peer Review File [file 41467_2020_20828_MOESM2_ESM.pdf]

**Editorial Note:** This manuscript has been previously reviewed at another journal that is not operating a transparent peer review scheme. The manuscript was considered suitable for publication without further review at *Nature Communications.*
